# Deep image features sensing with multilevel fusion for complex convolution neural networks & cross domain benchmarks

**DOI:** 10.1371/journal.pone.0317863

**Published:** 2025-03-18

**Authors:** Aiza Shabir, Khawaja Tehseen Ahmed, Arif Mahmood, Helena Garay, Luis Eduardo Prado González, Imran Ashraf

**Affiliations:** 1 Institute of Computer Science and Information Technology, The Women University Multan, Multan, Pakistan; 2 Department of Computer Science, Bahauddin Zakariya University, Multan, Pakistan; 3 Department of Computer Science & Information Technology, The Islamia University of Bahawalpur, Bahawalpur, Pakistan; 4 Universidad Europea del Atlántico, Santander, Spain; 5 Universidade Internacional do Cuanza, Cuito, Bié, Angola; 6 Universidad de La Romana, La Romana, República Dominicana; 7 Universidad Internacional Iberoamericana, Campeche, México; 8 Fundación Universitaria Internacional de Colombia, Bogotá, Colombia; 9 Department of Information and Communication Engineering, Yeungnam University, Gyeongsan, South Korea; Vellore Institute of Technology: VIT University, INDIA

## Abstract

Efficient image retrieval from a variety of datasets is crucial in today's digital world. Visual properties are represented using primitive image signatures in Content Based Image Retrieval (CBIR). Feature vectors are employed to classify images into predefined categories. This research presents a unique feature identification technique based on suppression to locate interest points by computing productive sum of pixel derivatives by computing the differentials for corner scores. Scale space interpolation is applied to define interest points by combining color features from spatially ordered L2 normalized coefficients with shape and object information. Object based feature vectors are formed using high variance coefficients to reduce the complexity and are converted into bag-of-visual-words (BoVW) for effective retrieval and ranking. The presented method encompass feature vectors for information synthesis and improves the discriminating strength of the retrieval system by extracting deep image features including primitive, spatial, and overlayed using multilayer fusion of Convolutional Neural Networks(CNNs). Extensive experimentation is performed on standard image datasets benchmarks, including ALOT, Cifar-10, Corel-10k, Tropical Fruits, and Zubud. These datasets cover wide range of categories including shape, color, texture, spatial, and complicated objects. Experimental results demonstrate considerable improvements in precision and recall rates, average retrieval precision and recall, and mean average precision and recall rates across various image semantic groups within versatile datasets. The integration of traditional feature extraction methods fusion with multilevel CNN advances image sensing and retrieval systems, promising more accurate and efficient image retrieval solutions.

## 1. Introduction

Digital media has become an essential part in modern life with advances in user-friendly applications, online data search, and document retrieval. Images are one of the most vital forms of digital media. Digital image has discrete or limited values for intensity. Digital image processing has made great advancements in the field of computer vision. Useful information is extracted and analyzed from both the query and retrieved set of images. A massive number of images are searched from a huge repository of databases over the Internet [1].

Image retrieval [2] is the process of searching images from different sources. Modern research trends lead towards exploration of efficient image indexing and searching techniques. Image retrieval can be text-based, semantic-based, and content-based [3]. Most of the proposed methods employ the content-based image retrieval [4].

Visual properties of primitive image features are employed in Content-Based Image retrieval (CBIR) [5]. Preprocessing extracts images from databases using these primitive features. Local features contain interest spots, spatial coordinates, and regions within images, while global features cover generic image qualities including shape, color, edges, and textures. A prevalent problem is the similarity in color histograms that makes color-oriented search difficult [6]. Shape-based image retrieval recognizes different shapes by highlighting areas and contours. Contour identification and subsequent boundary image information extraction is aided by methods like auto-regression, shape signatures, and polygon approximation [7].

Object recognition relies on describing shape geometry. Color features similarity makes it difficult to identify object exclusively. Color and form characteristics are combined to boost object detection and image retrieval. Object representation is enhanced by adding spatial information to colors. Corners and edges identification and computing pixel intensities are useful descriptors. Histograms show various color image rotations and scale invariants. Global features inadequately represent an image's geographical distribution and qualities. Their restricted application makes them vulnerable to image matching. Contrarily, local features based semantic gap reduction using interest point detection is favorable.

Enormous algorithms contributions including Harris [8], Hessian [9], affine invariant [10], and scale invariant [11] locate interest points. Local and global features are merged to identify objects and image contents. CBIR based systems exploit visual models like Bag-of-Words (BoW) [12] paradigm for data representation. CBIR based methods using BoW produce compact image features and achieve variance to image alterations [13].

Effective image retrieval and classification algorithms are being proposed by merging CBIR models to deep learning techniques [14]. Research trends focus on exploration of deep learning based effective techniques for effective images retrieval. Deep learning is the solution for asymmetry problem in feature extraction and representation. Modern deep learning based techniques use Convolutional Neural Networks (CNNs) for their adaptability to large scale datasets [15]. The CNNs based proposed methods [16] have shown improved performance results for image descriptions and retrieval. Image semantics are properly defined using CBIR in combination with deep learning. Conventional image representations rely on attributes and perceptions imposed by humans. Semantic gaps must be filled up properly. Deep features extracted thorough CNNs reduce the semantic gap between low-level information and human perceptions. CNN models created visual features efficiently exploit high-level information for translation. Several feature descriptors [17] are also applied in an analogous manner at various phases. Deep feature based image classification using CNNs provide effective learning with less data and improves performance for tiny image datasets.

Deep Convolutional Neural Networks (CNNs) also applied to transfer learning improve performance across several datasets by pre-training on one dataset [18]. They have outperformed other machine learning techniques for numerous applications. Deep learning-based CNNs paired with other image retrieval algorithms perform optimally for multiple tasks. Different structurally robust deep learning-based models yield competitive outcomes for a variety of image retrieval applications. AlexNet [19], VGG [20], ResNet [21], DenseNet [22], and GoogLeNet [23] are some trendy CNN models. Their creative methods and excellent results make them pertinent across a range of image retrieval applications.

Due to these recent developments in image processing and analysis, there have been a lot of developments in many different areas such as medical diagnosis and automatic security. Despite this, a major factor which is currently unresolved is the ability to create systems that could unify the operation of deep image sensing analysis and retrieval under single architecture. This integration plays a very vital role because it makes the different operations systems to be in harmony in processing and delivery of information. Moreover, due to the increasing numbers and variation of the size of the datasets, creating efficient image search scripts to cater for both, the large datasets as well as the small ones are proving to be central to the success of any application. Another important criterion is the availability of symmetric algorithms that could provide a high level of performance when working with versatile image benchmarks and that would be resistant to different datasets. Similarly, it is crucial to pay attention to colored and grayscale images with the same level of analysis stability at higher resolution as scans accumulate information for more accurate image interpretation. These issues are solved in this research by considering sophisticated strategies for the sensing and retrieval, stable algorithm development and image generalization for different scales. Major objective is to propose a more flexible and effective technique for modern image analysis and retrieval goals.

An enhanced algorithm based feature extraction technique intends to bring innovation to research. Local features are collected and analyzed using CNN architectures such as AlexNet, GoogLeNet, DenseNet, ResNet-101 [24], and Inception Net v2 [25]. These extracted features are improved using shapes, colors, background and foreground objects, and other spatial coordinate properties. The deep image contents acquired by factorizing, thresholding, and corner responses are revealed simultaneously based on straddling and autocorrelation. Image analysis involves signature altering and datasets harmonization. Image content analysis is enhanced by combining image indexing and categorization. Characteristics with signatures are extracted through deep learning-based analysis. Extracted features are then integrated to architectural algorithm's elaborations, suitable coefficients, and parallel joints of color displacements. Deep learned features for both colored and gray scale images are integrated to generate efficient and compact image feature vectors. CNNs and algorithmic channelizing is employed to generate CNN based signatures. Feature vectors are merged with CNN signatures to achieve consistent variety of categories for datasets and semantic groups. Experimentation is performed by integrating the presented approach with various CNN designs as DenseNet, AlexNet, InceptionNet v2, ResNet-101 and GoogLeNet. Spatial head integration produced from color channels enables architectural bonding. Color coefficients are integrated with L1 and L2 normalization [26] at RGB color channels. Deep learned features [27] and compact feature vectors are created by fusing signatures with coefficients. The presented approach is further evaluated on a number of datasets including ALOT [28], Cifar-10 [29], Corel-10K [30], Tropical Fruits [31], and Zubud [32].Image searching for database tiny, large, complex, or mimicked images is performed. The inclusion of BoW architecture increases the usability and effectiveness of the presented technique and provides proficient classification and retrieval of images.

Contributions of the presented approach are following:

- Provides fusion based image retrieval method using CNN signatures for gray scale and colored images feature extraction with shape, color and texture features.
- An efficient feature detection method is provided using sampling, smoothing, filtering, suppression, scaling and placement techniques that results information related to image local features.
- Dense deep learned patterns compatibility is achieved with global signatures, and Cross domain mapping is offered for state of the art benchmarks datasets.
- Heterogeneous structure CNN fusion is provided with primitive features and color channels that work on both 255 levels of gray scale images and for RGB channels.
- Multi structural formation for complex datasets is provided with enhanced accuracy in results, enhanced CNN proficiency achieved and images salient features are coupled with CNN architecture.
- Creates a fusion for basic features vectors and CNN to the bag of visual words framework for image indexing and ranking and retrieval.

## 2. Literature review

Modern research trends mainly focus on finding better pattern recognition and features extraction techniques to improve CBIR performance. Effective Methods using machine learning and deep learning based methods are being proposed to provide efficient image retrieval. A method for image description using deep learning is presented [33]. AlexNet CNN is applied for image retrieval and classification using Local Binary Pattern (LBP) descriptors and combines it to Histogram of Oriented Gradients (HOG). Image dimensions reduction is achieved through Principal Component Analysis (PCA) method. Datasets Corel-1000, OT, and FP are employed in experimentation to provide performance comparison with existing methods. Results showed improved accuracy rates and better mean of Average Precision (mAP) and also reduced the computational overhead.

A detection method for Alzheimer's disease using CNN is presented [34].Early stage detection is achieved using 3D auto encoder techniques, 3D CNN and 3D Capsule Networks (CapsNets).CapsNets enables efficient learning to methods and works well on small datasets. Combining these techniques provides better performance than a standalone deep learning-based CNN. The presented method yields superior image classification results. A novel approach [35] for feature extraction at different layers is presented. Feature extraction is performed by introducing a mapping function that highlights the effectiveness of similarity at a lower layer. A similarity check is conducted for the query image to its nearest neighbors with similar semantics. Experimentation results demonstrate the effectiveness of the presented method across different retrieval benchmarks. A bi linear architecture for feature extraction is presented using CNN [36]. Feature extraction is performed through two CNN architectures working in parallel. Features are directly extracted at various scales and locations of images on different convolution layers. The deep learning-based CNN architecture is pre-trained on a generic image datasets, and bi linear pooling is utilized to reduce image feature dimensions. Back propagation is employed for final training, enabling the architecture to learn various important parameters for image retrieval. Experimentation on three standard datasets benchmarks shows that the presented method outperforms other approaches in terms of performance, time efficiency, and storage memory requirements. Another method [37] introduced a new model for CNN that can be employed in indexing, extraction, and retrieval of images. Low-level descriptors are generated through Random Maclaurin projection, and standard image datasets are employed to evaluate the image descriptor efficiency. The presented architecture's scalability is evaluated on a dataset containing one million images, with performance assessed based on accuracy, speed, and storage requirements. GPU kernels are also evaluated for their retrieval and processing performance.

A simple framework proposes a CNN using deep learning and Support Vector Machine (SVM) for CBIR [38]. Empirical studies show that the presented method can improve CBIR performance. An architecture network is presented [39], which is end-to-end trained and uses CNN to solve image related problems. It addresses image retrieval by employing learning based on the representation of images. A new method using the triplet mining method is presented, providing local pooling on multiple scales. Experimentation on three standard datasets demonstrates better performance. An approach [40] is presented for the representation of features based on various approaches to deep learning. The presented approach works on hand-crafted feature representations and categorizes editorial-based images into six sets of classes. Experimental results show better performance for these combined sets of features across various classes.

Mostly CBIR techniques are based on image features at a lower level. A novel algorithm is presented [41] that work in two stages. At the first stage, feature extraction is performed using CNN, and an Ensemble Learning (EL) model is employed to add novelty to the CNN-based approach. Comparison results show better performance for the presented algorithm in terms of image retrieval. A technique for the quality assessment of retrieved images is presented [42]. A technique called CNN-DBN uses CNN models for feature extraction and builds a model based on Deep Belief Network (DBN) to assess quality. Experimental evaluation is designed to assess the method’s ability and performance. An image retrieval model [43] is presented that uses triplet CNN for feature learning using certain criteria for similarity metrics. It also proposes methods to improve CBIR task performance. The model works without using any activation function to preserve feature information and combines features received from multiple layers for the image retrieval task. Experimentation results show the effectiveness of the presented model for CBIR. The CNN-based feature extraction technique [44] combines computer vision approaches with CNN. Lower-level features are extracted using computer vision approaches, while high-level features from images are extracted using CNN. Increasing the number of layers increases the training time, and comparison is made between results obtained from different layers of CBIR and other computer vision-based techniques. A CNN-based method [45] for image feature extraction using Euclidean distance is presented. Image features are extracted for both the query image and the stored images, and performance evaluation is done through precision rates. Image classification results are improved through another presented technique [46]. New layers are added to the network, and an algorithm for feature extraction is presented. Image retrieval is performed using CNN-based representations. A Principal Component Analysis-based method is presented [47], utilizing deep neural networks for the classification of images. A method based on symmetry findings using scoring with neighboring points is presented [48] for deeply learned features through smoothing and standard deviation methods.

A fusion-based new method using the ResNet, VGG, and GoogLeNet architectures is presented [49]. Image classification is achieved through Random Forest and SVM methods, with experimentation conducted using the Stanford 40 actions dateset to obtain accurate results. The deep learning-based method presented [50] is based on five stages of image pre-processing, pre-trained CNN, semantic segmentation, query analysis, and image retrieval, utilizing the NTCIR13 Lifelog-2 dateset for testing the methodology. Deep neural networks (DNN) are employed for image stemming, recognizing objects using AlexNet and GoogLeNet architectures, and scene classification through Places 365 using AlexNet, ResNet, GoogLeNet, and VGG architectures. The CRIB-based method presented [51] uses visual features such as edges, shapes, color, and texture for processing. Image classification is performed using CNN, which uses cosine for image retrieval. An efficient CNN-based method [52] is presented for sparse representation, including a new method for detailed feature extraction, providing accurate results for image retrieval. These methods are also efficient and time-saving for sparse representations. A framework proposed [53] for noise reduction that uses histogram equalization. Feature extraction is performed by Gray Level Co-occurrence Matrix (GLCM), Hierarchal and Fuzzy c- Means (HFCM) algorithm is applied to match similarity computations and Deep Learning based Enhanced Convolution Neural Network (DLECNN) algorithm is employed for image retrieval. The proposed method shown improved results related to accuracy, precision, f-measure, recall and reduced complexity. It also provided better retrieval for the query images.

In current research trends, more emphasis has been placed on optimizing synchronization techniques for complicated neural networks employing a variety of mathematical models. In [54] focus is on the event-triggered synchronization of coupled neural networks with reaction–diffusion terms where an event-triggered controller is employed to update the weights at event instants in accordance to certain trigger criteria. This strategy has the advantage of lowering the communication burden in control systems more than the continuous control approach. By means of inequality techniques and the new designed controller, the study offer the criteria for the event-triggered synchronization and the numerical examples verify the theoretical finding. Likewise, in [55] an adaptive synchronization has been studied in FOUCVCNNs using non-decomposition method for fractional order systems. Here, the FOUCVCNNs model is derived to preserve its complex-valued structure and has not been further divided into real entities. The paper presents an adaptive controller to minimize the control costs, referencing to fractional Lyapunov theory, 1-norm analysis, and inequalities and providing synchronization criteria using the Lyapunov equation. Computational simulations support these results. Further, in [56] there are new developments that go beyond the integer-order kinetics to describe tumor growth and incorporate fractional-order phenomena in cellular response. This model describes the interaction of cancer stem cells and non-stem cancer cells using a system of coupled nonlinear integrodifferential equations. Using the properties of Mittag-Leffler functions and fixed-point theory, the authors prove the existence and boundedness of the solutions. The finite difference scheme is used to explain the efficiency of the model through the consideration of several numerical examples Simulations provide information on cancer development, and the tumor growth paradox linked with cancer stem cells.

## 3. Proposed methodology

The presented method comprises different stages for deep image retrieval. The first phase is to describe and explore image key points for feature detection. Descriptor formation and matching is performed to find next-level details for the explored features. Color Channelizing is applied to find the color coefficients for the colored images. Color components denote the color features of the images. Fusion of CNN-based networks is applied to extract the deep-learned features. Finally, the features collated from the stages are collected through BOVW. Image indexing and ranking is applied for relevant and efficient image retrieval. Modularity is the key feature of this presented method. It combines various image detectors with the key point descriptors. The following section provides an overview of the main components of the presented method:

### 3.1. Detection and depiction of keypoints

The first step is to convert the colored images to gray scale levels between 0 and 255. Major benefit of gray scale image conversion is the image noise reduction. Noise reduction in gray scale images is achieved through varying intensity levels for black and white. High and low level intensity creates a color combination for black and white. Key points [57] are the main points of interest in any image that provide valuable information for image retrieval. Efficient feature detection is the key to efficient image retrieval systems. The presented method provides a combination of effective approaches for detecting salient regions of interest from a huge repository of images.

### 3.2. Scale axis discretization

**Fig 1:**
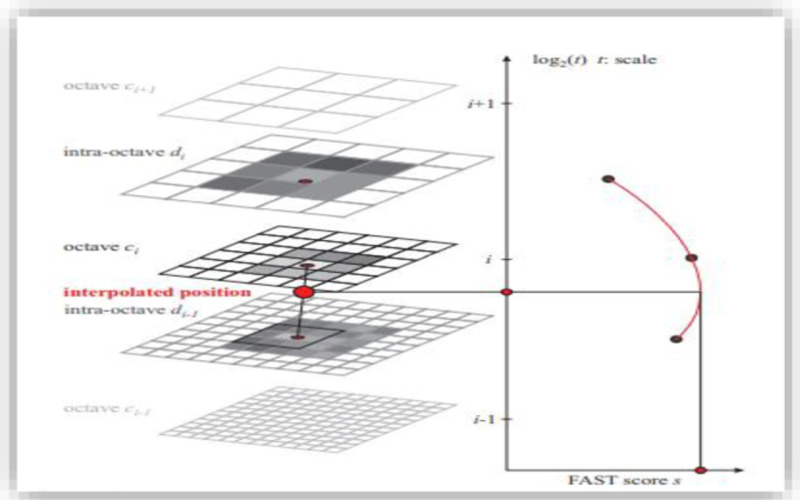
Scale space key point’s detection [55].

Scale invariance is crucial for high-quality key points detection. Scale invariance provides detection and exploration of local features. Scale space defines the areas of interest for image saliency. Local maxima is searched on this scale axis as well as in the local image plane using the FAST [58] scores. The presented method estimates these key points using the continuous space for scales. Scales axis is discretized at the coarse level of intervals as described in Fig 1.

### 3.3. Objects recognition and description using BRISK

**Fig 2:**
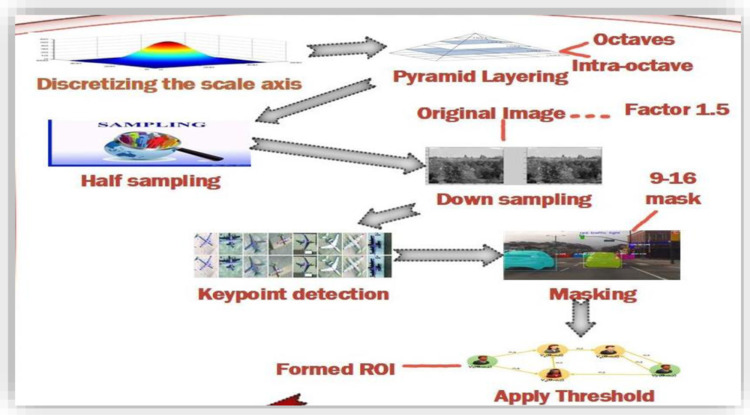
Pyramid layering and image sampling.

The sampling frequency is selected on the basis of the right sampling criteria. Instantaneous values from the continuous signals are received from Analog to Digital convertor. These sample values are based on instant sampling of the signals. High-frequency samples are considered for comparison with the last seen changes in the signals. Sampled signals are the samples taken from the low frequency and compared with the signals of high frequency. The BRISK framework [59] works on the formulation of a pyramid for the scale-spaces. This pyramid is based on octaves and intra-octaves that help to achieve half sampling for the basic image. Fig 2 shows the pyramid layering and image sampling features. Intra octaves are located in between the other octave layers. The original image is down-sampled by a factor of 1.5 to obtain the first intra-octave. Other remaining intra-octave layers are simply achieved through half sampling.

The image down sampling is performed for resolution reduction in the spatial domain within 2D representation. Down sampling reduces image storing and transmission requirements. While up sampling is the increase in spatial resolution for an image 2D representation.

**Table:** 

**Algorithm 1**: Detection of key points
1: Image=IMG 2: P=Pyramid 3: Key points=KP, FAST key points=KP_F, Harris key points=KP_H, Refined key points=KP_R, Final points =KP_F 4: Gaussian blur based Pyramid generation on various σ values P = {IMG _1 _, IMG _2 _, IMG _3 _,…., IMGn} 5: For each level scale in P *i *=1 to *n * 6: KP_F= FAST_D (IMG _*i *_) 7: Response Score= KP_H (KP_F) 8: Refined points= KP_R (KP_H, IMG _*i *_) 9: KP_F _*i *_= Suppression (KP_R) 10: Merge all scale level key points: KP_F= ⋃KP_F _*i *_ 11: End for

### 3.4. Image shape formation and suppression

Masking is applied to explore important points in any shape of the image. 9-16 masking is applied in the method that requires a minimum of 9 pixels at consecutive levels and 16 pixels in a circle position that forms a bright or dark side for the points in comparison to the center pixel point. A FAST detector initially with 9 to 16 separate masking with each octave applied, then to the intra octaves. Lastly, the same threshold value T is applied to detect the important regions in the images. Thresholding is the last operator point in the area of interest. This value is specified pixel location within the specified range that provides image object identification with their known brightness range. Object brightness is also an important factor in the object identification.

Uniform Thresholding and adaptive thresholding are two major types of thresholding. Uniform thresholding separates and identifies pixel levels between black and white. Brightness is set to white for an original eye image pixels above 160 levels, and pixels below 160 levels are black. Facial skin pertained to parts separated from the image background and other bright areas of the face are also separated from hair and eyes. Thresholding provides an efficient isolation and separation of areas of interest in the images.

### 3.5. Edges and corner responses

**Fig 3:**
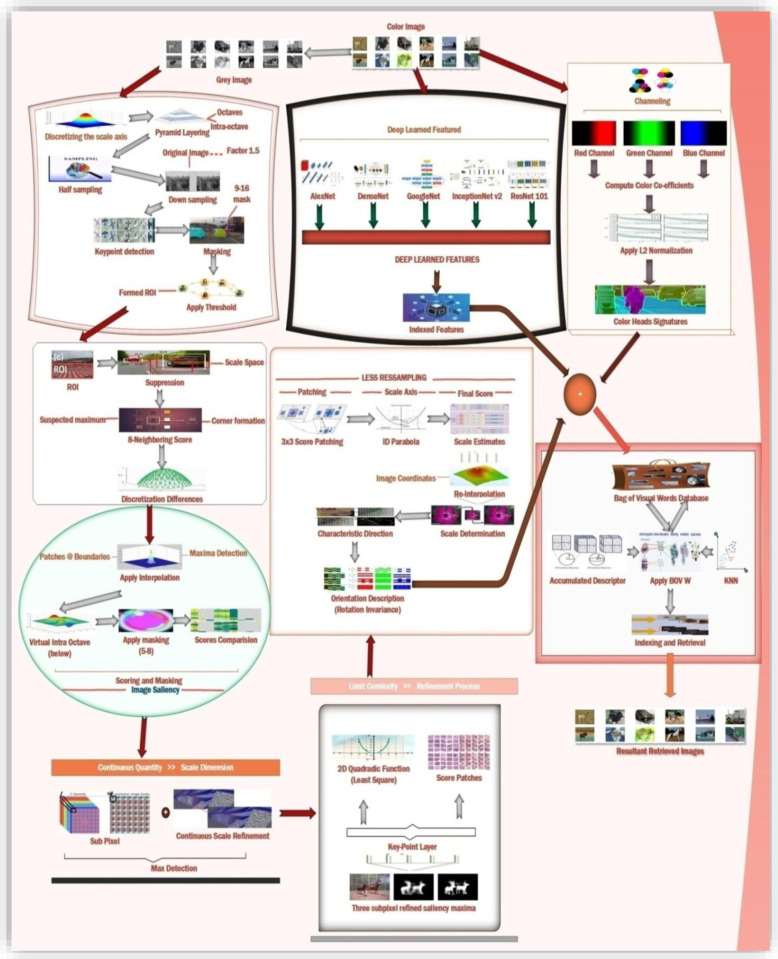
Image region of interest.

Edges are considered another important factor for exploring image features. Suppression is the technique for finding the edges from the query image. This method focuses on the relative points that lie at the ridge top of data edges and the remaining points are suppressed. It gives the output in the form of thin lines from edge points. Edges are also formed by finding the location of each single edge point. Gaussian smoothing is combined with first-order differentiation to form the Gaussian template. It provides a smoothed image with the edges formed through the ridge of the image data. Image can be convolved with the operator to find edges at the exact and correct points. Fig 3 shows the methods used to find edges and corner responses in images. This convolution results in the first derivative to the edges in their normal direction. The maximum function results in edge location that is the peak value of the edge data with the sharp image gradient.

Operator G_x_ is formed through the Gaussian function G derivative of in the normal direction denoted by x [60].


$${\text{G}}_{x}=\frac{\partial \text{g}}{\partial {\text{x}}_{⊥}}$$ (1)

Where x is the estimation from Gaussian function g first order derivative that is convolved with the image I and represented as:


$${\text{x}}_{⊥=\frac{\overset{"}{\nabla }\left(\text{I*g}\right)}{\int \overset{"}{\nabla }\left(\text{I*g}\right)}}$$ (2)

The maximum point for the Gaussian function Gx formed through convolution with the image is the true edge point location. This is formed when the value for differential is zero:


$$\frac{\partial \left({\text{G}}_{x}*\text{I}\right)}{\partial {\text{x}}_{⊥}}=0$$ (3)

Substitution of Eqn. (1) in Eqn. 3 [60]:


$$\frac{\partial^{2}\left(\text{G}*\text{I}\right)}{\partial {\text{x}}_{{⊥}^{2}}}=0$$ (4)

This suppression is equivalent to the peak values that are perpendicular to the edge. It thins the operator for edge detection to find the right place without having multi-point edges. It also contains minimal noise in the responses.

Points of interest that make the regions applied to suppress the non-maxima in the scale space. These key points need to fulfill the 8 neighbors FAST scores maximum condition in the relative layer. The maximum value of the threshold considered as a corner point is defined by these scores. The second condition for suppression is that the scores for the upper and lower layers need to be lower than these points values. Patches are divided into equal sizes of squares and the side size is determined by the 2-pixel values within the layer with the expected maximum.

### 3.6. Applying interpolation for image enhancement

Interpolation is required at the patch boundaries, as these layers at the neighboring side are defined by using variant discretization. Maxima are detected at the initial scale axis that is the intra-octaves and the below-level scores. After finding maxima, 5-8 masks applied to the interest point values. Scores for the above patch for this case may not be lower than the octave points of interest. Image saliency is considered as continuous quantity along the scales dimensions across that image. For scale dimension-based image saliency, each maxima value detect is refined by the scale continuous values and with the sub-pixels.

**Fig 4:**
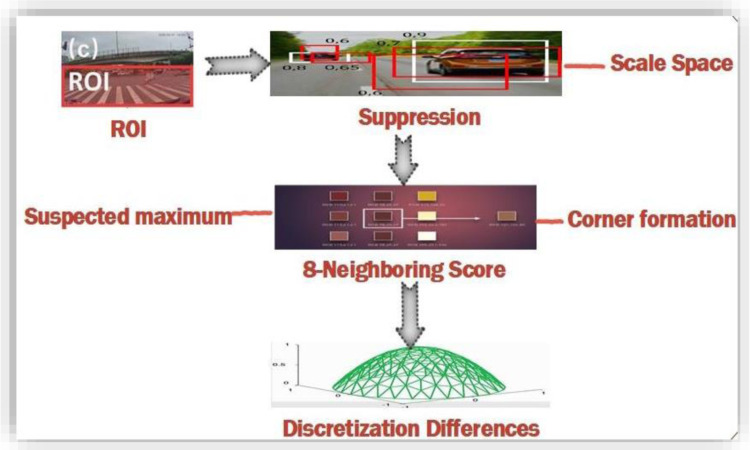
Presented Methodology.

**Fig 5:**
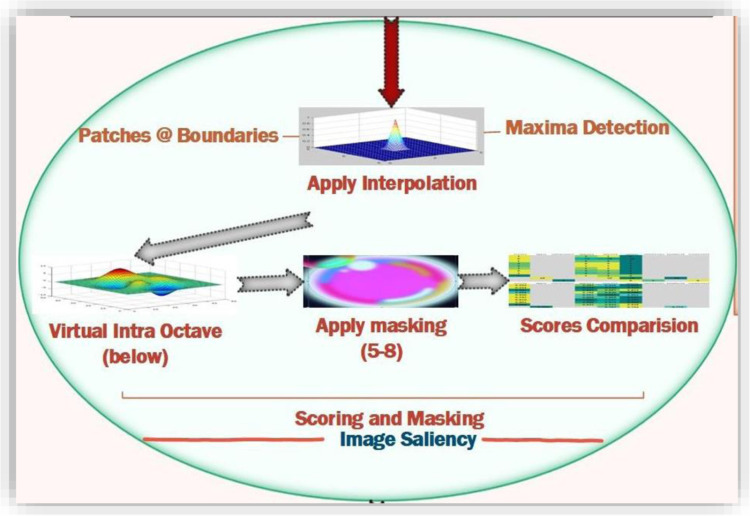
Interpolation and image Saliency.

Fig 4 shows the methodology for the presented method. Least squares are fitted for the function of 2D quadrates to each of obtained patches scores. Three scores for the patches are obtained, one from the layer of the key points and the other from the above and lower layers. These three scores contribute to the refined valued saliency maxima in three sub-pixels. 3x3 patching scores are considered at each layer to avoid the risks of resampling. 1D parabola across the axis is defined using these refined valued scores that contribute towards the final estimated scores and the estimated scale point to the maximum. Finally, the presented method re-interpolates the coordinated image among the layer patches along the scale. Fig 5 represents the saliency and masking offered in presented method.

**Table:** 

**Algorithm 2:** Descriptor formation for Keypoints
1: Key points= KP, Image=IMG, Descriptor=D, Local patch=LP, Intensity Differences=ID, Encoded Descriptor=ED 2: For each k in KP 3: Ø = Orientation Computation (k, IMG) 4: LP= Patch Extraction (k, IMG) 5: ΔIDk= Compute Intensity Differences (LPk) 6: Quantization of Intensity differences ◊ ED= Descriptor Encoding (ΔIDk) 7: D = {D1, D2, D3, …,Dm}

**Fig 6:**
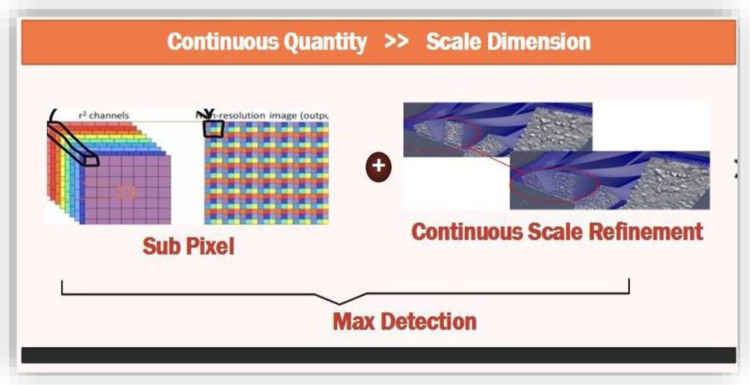
Maximum detection.

Key points are formed through a refined set of sub-pixels in the image and the continuous values for scales as shown in Fig 6. A binary string is formed through the concatenation of comparison test results for brightness. Direction characteristics for key points are identified to achieve invariance for rotations and normalized orientations. This invariance for rotations is very important to achieve robustness. The main concern in selecting these comparisons for brightness is to maximize the descriptiveness.

### 3.7. Pattern sampling and rotations using DAISY

**Fig 7:**
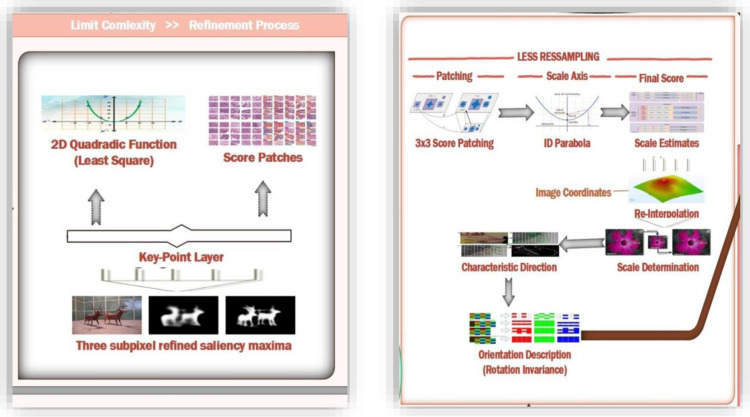
Refinement Process and Orientation.

The presented approach uses pattern for sampling the neighboring points to the key point. The Pattern shown in Fig 7 is defined through N locations making a circle around the central key point. This pattern provides matching of denses and resembles the DAISY descriptor [61].

Gaussian smoothing with standard deviation applied to avoid the effects of aliasing. Standard deviation is performed, σi with the proportion to the respective circular points distances. Scale and position pattern according to the specific image key point is considered as S. Sampling point for the (S − 1)/2 is denoted by the pair (x_i_,x_p_). Intensity values at the sampling points are V (x_i_, σ_i_) and V(xj, σj). These intensities values estimate local gradients that are denoted by Gv(x_i_,x_j_) [62].Where:


$$\text{Gv}\left({\text{x}}_{i}{\text{, x}}_{\text{j}}\right)\text{= }\left({\text{x}}_{j}-{\text{x}}_{\text{i}}\right)\frac{\text{V}\left({\text{x}}_{j}{\text{, s}}_{\text{j}}\right)+\text{V}\left({\text{x}}_{i}{\text{, s}}_{\text{i}}\right)}{{\text{xj-xi}}^{\text{2}}}$$ (5)

Consider a set A for all the sampling key pints pairs:


$$\text{A}=\left\{\left({\text{x}}_{i}{\text{,x}}_{\text{j}}\right)\in {\text{R}}^{2}\times {\text{R}}^{2}|\text{i}<\text{S}\wedge \text{j}<\text{i}\wedge \text{i},\text{j}\in \text{S}\right\}$$ (6)

Two subsets for distances are formed; one for pairs at shorter distances D and the other is the long-distance Z:


$$\text{D}=\{\left({\text{x}}_{i}{\text{, x}}_{\text{j}}\right)\in \text{A }|<\text{δmax}\}\subseteq \text{A}$$ (7)

$$\text{Z}=\{({\text{x}}_{i}{\text{, x}}_{\text{j}})\in \text{A}|\text{<δmin}\}\subseteq \text{A}$$ (8)

**Table:** 

**Algorithm 3:** Descriptor Matching
1: Descriptors=D _1 _, D _2 _, Matched points Result= MP, Match Verification=Match _v _, Threshold=T _v _ 2: For each descriptor *ds *_*1 *_ * in D *_*1 *_ Ds _match _= Match Descriptor (ds _1 _, D _2 _) 3: For each (ds _1 _, ds _match _) Match _v _= Match Verify (ds _1 _, ds _match _, D _2 _) 4: Apply Threshold to matched filters ◊Threshold Application (Match _v _,T _v _)

Threshold values for δmin and δmax set according to the scales. By using the iteration for the pair point values for Z, the overall pattern direction Y is characterized. The long-distance pairs for computing the gradient values locally [62]:


$$g=\left(\begin{array}{c} g_{x}\\ g_{y} \end{array}\right)=\frac{1}{Z}.\underset{\left(x_{i},x_{j}\right)\in Z}{\sum }g\left(x_{i},x_{j}\right)$$ (9)

### 3.8. Color-head signatures description and formation

Color images also use pixel intensity to store images. Colored images use three intensities for color representations in images. RGB model is based on three colors red, green, and blue [63]. Color segment is represented in two ways. Each color value is linked to an integer value associated with each pixel that indexes a table showing intensity values for each color component. The index values retrieve exact information about the actual color whose value is to be represented in the pixel displayed or being processed. This method of using tables is called the image palette. The color display is performed using the color mapping from the pixels to the table values. Color mapping reduce the storage requirements that save just one single plane for the image representation and the associated palette. The disadvantage of using this technique is that color collection is reduced. So, the alternative approach for color representation is to use multiple color image planes that store each pixel's color components separately. Color specification is called a true color method. True color representation provides more accurate color specifications and covers more colors as well.

**Fig 8:**
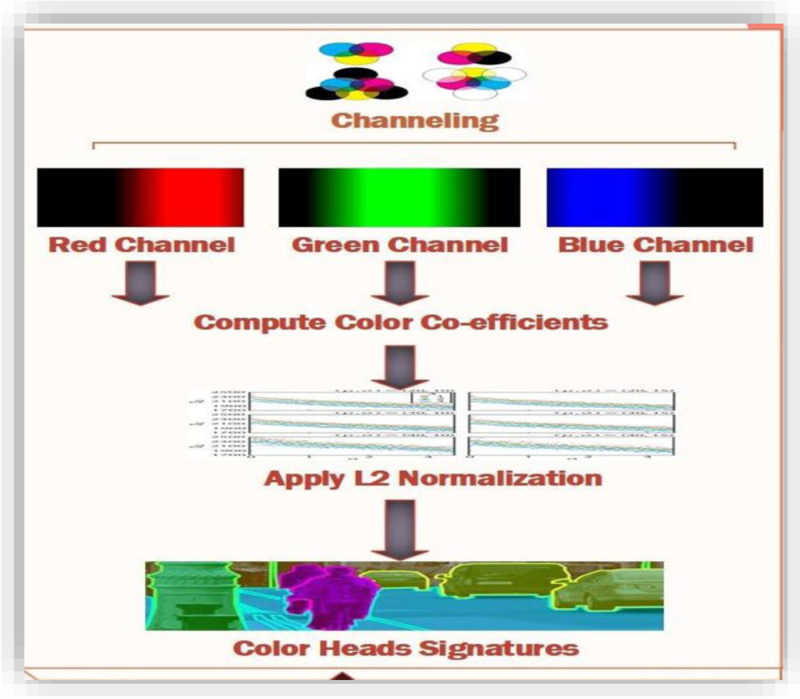
RGB Channeling.

The presented method deals with both gray scale and colored images as shown in Fig 8. Gray scale images in the presented method efficiently extract the features from the query image and perform analysis. RGB components represented as channels are also features. These channels hold basic color information. The presented method uses color mapping to explore deep features. The coupling of color-based information with grey values is very useful in finding image features. The color feature provides object information. Spatial coordinates in collaboration with color features resolve similarities among objects in any image. Color channeling can help attain better results in precision and recall.

Image is segmented based on information obtained from various scenes and objects of an image. Segmentation process applied using labels and scenes require processing on specific pixel related to these objects in the colored images. Processing the segmentation on the colored image is a complex task to accomplish. To solve this problem L2 normalization is applied to the colored components of the image. L2 normalization is applied to blur the cross across-pointing in the color coefficients of the objects.

### 3.9. CNN based Signatures Formation

**Fig 9:**
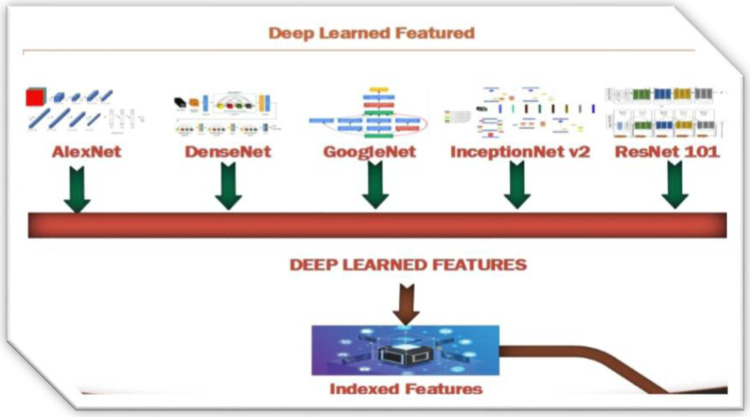
Deep learned features.

The presented method provides compact and efficient image feature vectors represented in Fig 9. These deeply learned features provide integration for colored and grey-level images. That is combined with CNN signatures formed through Convolutional Neural Network models. Algorithmic channelizing is integrated to achieve uniform diversity for datasets and semantic groups [64]. Experimentation is performed to get a parallel response for merging the presented method with other CNN architectures like DenseNet, AlexNet, GoogLeNet, ResNet-101, and InceptionNet v2. Architectural bonding achieved through spatial heads integration that is generated from color channels. Compact feature vectors are formed by incorporating coefficients with signatures. Deeply learned features search all types of images form databases including tiny or large or even complex or mimicked images.

DenseNet is a CNN-based model that uses dense blocks and dense connection layers. Each dense block is considered as a separate layer in the network. The bottleneck layer in each dense block contains 11 convolution layers for input features reduction and 33 more convolution layers. All layers are interconnected and each layer gets input from the upper layers and passes this information through maps of features to all other subsequent layers presented in the network. Hence each layer in the network is connected and receives relative features information from the former layers. It makes DenseNet Architecture thinner and complex network model. Batch Normalization (BN) and ReLU layers with 3x3 convolutions depict the output features from multiple channels.

Applying AlexNet, InceptionNet v2, GoogleNET, ResNet-101and DenseNet CNN-based network architectures with the presented method enhanced the quality and accuracy of objects detection and recognition. Feature vectors [65] induced to these CNN models generate the deep learned signatures that are more powerful and signified features for images.

### 3.10. Applying BoVW for Image Indexing and Ranking

Bag-of-Visual-Words (BoVW) architecture provides image indexing and retrieval. Each image is represented as a linear vector in this Visual BoW architecture. Ianthe first stage, this model defines the controls as Scale Invariant Features Transform (SIFT) [66] for the descriptor of image local features. Then the comparison is performed at each of the single vector for the image using scores for the dissimilarity check that gives more distinct features. Local features descriptor based on SIFT represents the patches in the form of vectors having numeric values.

SIFT descriptor collects 128 bits vectors having equal size of dimensions and represents them in linear form that makes an effective and compact image representation [67]. Visual words are created and represented in the form of histograms. These histograms create inverted image indexes that provide an efficient retrieval of the images. Each index generated by the histograms represents a single visual word. Each part of the object information contains details related to various aspects of features like texture, shape, and color. These visual words contain information based on changes in signal intensities caused by filters and other low-level features and also creates the list of image identity map terms [68].

**Fig 10:**
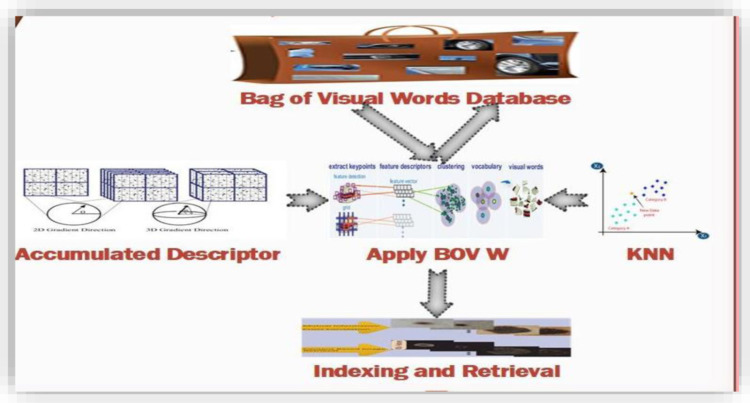
Bag of words and image indexing.

The last step shown in Fig 10 is to count the total number of visual words obtained through the BoVW model to perform ranking for the query and indexed images. Top-level rank is assigned to the shared words of the image having highest frequency in numbers. This model does not capture the co-occurrence between the visual words, the spatial information, or the specific location information related to the images [69]. Color feature extraction is employed in the presented method to extract specific spatial information for features.

## 4. Databases

In the evolving domains of computer vision and machine learning, access to high-quality datasets is of paramount importance for the development and evaluation of advanced algorithms and models [70]. These datasets, noted for their ability to present diverse and real-world scenarios, are invaluable resources for researchers and practitioners. Experimentation is performed on ALOT, CIFAR-10, Corel-10k, Tropical Fruits, and Zubud databases. These datasets provide efficient image classification and recognition.

### 4.1. ALOT

**Fig 11:**
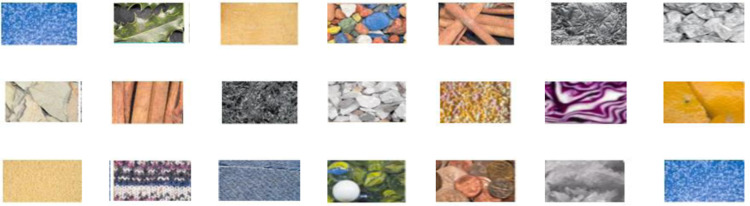
ALOT Datasets sample images [28].

ALOT (A Large Outdoor Text Dataset) datasets provide detection and reorganization of text scenes. It provides a vast collection of images from outdoors places or public areas like parks, streets and other common settings. ALOT create and assess the algorithms that categorize and recognize the text related images. ALOT database main categories are basically the signs of streets, fronts of stores, billboards, labels of products and other text related to environment. Researchers attain improved performance for the algorithms by using ALOT based text data. ALOT provides better detection for text, recognizes the signs of streets, and to analyze the documents. ALOT dataset is applied in text related research and performs well on illumination changing, weather forecasting, and text orientations. Fig 11 shows the sample images for ALOT dataset.

### 4.2. Cifar-10

**Fig 12:**
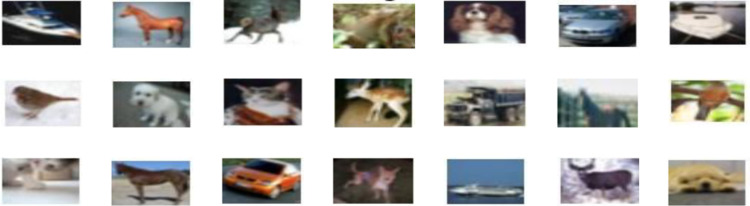
Cifar-10 Datasets sample images [29].

Cifar-10 is another trendy dataset that provides image classification. It comprises on ten different classes having 60,000 color images of size 32x32. The dataset employed in applications designed for image classification to assess standard machine learning and deep learning methods. Cifar-10 dataset contain ten category sets of Ships, Frogs, Birds, Trucks, Cats, Deer, Dogs, Airplanes, Horses, and Automobiles. It tests algorithms designed for image classification. Cifar-10 dataset provides fast testing and evaluates the models effectively to categorize and represent the images. Fig 12 shows the sample images for Cifar-10 dataset.

### 4.3. Corel-10k

**Fig 13:**
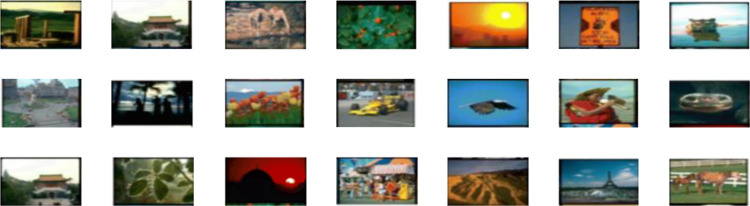
Corel-10k Datasets sample images [30].

Corel-10k dataset is another useful dataset for image classification and representation. This dataset has 10,000 images of 100 different categories. These images are based on various domains.Corel-10k deals with noisy data and imprecise category problems. These 100 categories in Corel-10k datasets are based on Music, Animals, Cars, Sports, People, Architecture, Food, Flowers, Art, Landscapes, and Electronics. Corel-10k is effective dataset for more complicated and diverse set of categorization. Diverse set of categories makes it suitable and robust dataset to test various classification methods. Fig 13 shows the sample images for Corel-10k dataset.

### 4.4. Tropical-Fruits

**Fig 14:**
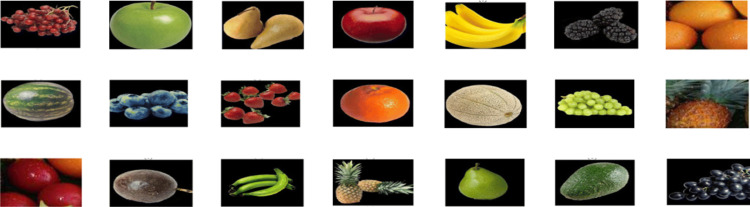
Tropical-Fruits Datasets sample images [31].

Tropical-Fruits dataset identifies and evaluates the quality of fruits. The dataset comprises on a wide range of categories for tropical fruits of various environments. It is mainly employed in agriculture based research. Some categories of Tropical-Fruits datasets are Sliced Fruits, Citrus Fruits, Apples, Exotic Tropical Fruits, Market Displays and Bananas. Fruit identification and classification methods are created and tested using this dataset. Fruits identification based methods are very vitalinsalient applications designed for agricultural and their quality assessment. Diverse set of fruit types makes it a suitable dataset for conducting research on fruits images recognition. Fig 14 shows the sample images for Tropical-Fruits dataset.

### 4.5. Zubud

**Fig 15:**
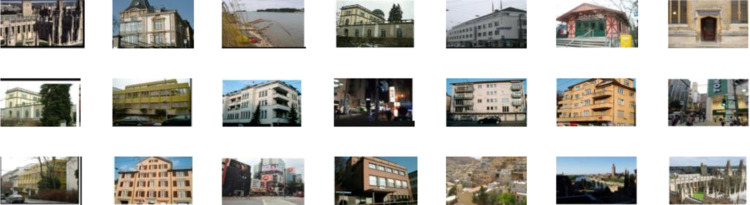
Zubud Datasets sample images [32].

Indoor scenes classification is basically provided through a database called Zubud. It has 11,000 images in the database that are divided into 11 different classes for interior scenes. Zubud is employed for smart homes technologies. It mainly provides recognition of scenes and navigates the indoors. Bar, Living Room, Kitchen, Office, Bedroom, Kids Room, Bathroom, Restaurant, Clothes Store, Gym, and Bookstore are some of the Zubud categories. Zubud train and assess models that identify and categorize scenes inside of enclosed environments in the domains of robotics, smart homes, and indoor navigation. Zubud offers a controlled environment for investigating indoor scene recognition and classification that makes it crucial component for practical applications in these fields. Fig 15 shows the sample images for Zubud dataset.

## 5. Results and Discussions

The presented innovative approach for image retrieval is evaluated through a series of criteria that systematically evaluate recall, precision, and overall efficiency. These measures are crucial for analyzing method's effectiveness in comparison to five common benchmarks. The metrics along with the corresponding mathematical expressions are:

Precision quantifies the percentage of relevant results among the ones that were obtained. The calculation involves dividing the total number of matched, relevant images (R) by the total number of retrieved images (S) [71].


$$\text{Precision}=\frac{\sum R}{\sum S}$$ (10)

The Average Precision (AP) is measured at each retrieval cycle as the level of precision for every image class (C). To calculate the results, each category precision values are added and then the sum is divided by the total number of iterations [71].


$$\text{AP}=\frac{\sum CiP}{\text{Total}\mathrm{Iterations}}$$ (11)

Average Retrieval Precision (ARP) provides comprehensive perceptive for precision. It is calculated using AP rates for each image in that class relating to total of all the classes. It contains the precision of all categories and are added gradually to the first class [71].


$$\text{ARP}=\frac{\sum CiAP}{\text{Total}\mathrm{Categories}}$$ (12)

Recall is an important metric for effective retrieval evaluations. It is the ratio of relevant and retrieved results to the related results. It is calculated as the sum of retrieved and relevant results and then divided by related results [72].


$$\text{Recall}=\frac{\sum S+\sum R}{\sum R}$$ (13)

Average Recall (AR) is the recall in every image class (C) over numerous iterations. It quantifies the value of recall within each class [72].


$$\text{AR}=\frac{\sum CiR}{\text{Total}\mathrm{Iterations}}$$ (14)

Average Retrieval recollection (ARR) is similar to ARP for the precision. ARR provides a broader level of recall evaluation.AR rate is measured for each image class in relation to the sum of all of the categories and values for recall at each category are gradually added to the first category [72].


$$\text{ARR}=\frac{\sum \text{CiAR}}{\text{Total}\mathrm{Categories}}$$ (15)

AP values are summed up for each category and result is divided by the total number of categories to calculate Mean Average Precision (MAP). MAP provides a broader level view for precision performance [71].


$$\text{Mean Average Precision =}\frac{\sum AP@C}{\text{Total}\mathrm{Categories}}$$ (16)

Mean Average memory (MAR) provides a complete evaluation of recall performance. It is calculated by summing up AR values for each category and then dividing the results by the total number of categories [71].


$$\text{Mean Average Recall}=\frac{\sum AR@C}{\text{Total}\mathrm{Categories}}$$ (17)

F-measure also called the composite metric evaluates the overall performance of retrieval. It is calculated by computing the harmonic mean of precision and recall for each category of images [73].


$$\text{F-measure =}\frac{\text{2*}\left(\text{V*U}\right)}{\left(\text{V+U}\right)}$$ (18)

The image retrieval time for proposed method varies, ranging from approximately 0.4 to 2.35 seconds. This variation is mainly subjective to the factors of image dimensions and size of the dataset. Experimentation's were conducted on a core-i7 processor running at 2.5 GHz with 8GB of RAM.“

Performance evaluation of the presented method is completed through various datasets. Experiments are conducted on different datasets, including ALOT, Tropical-Fruits, Zubud, Cifar-10, Corel-10k to evaluate how effectively presented approach handles complex images. The presented method along with multi level fusion on CNNs consistently achieves higher average precision results for complex images.

### 5.1. Experimentation on ALOT

The ALOT dataset is widely recognized for its intricate structure and is employed as a rigorous benchmark for image classification, particularly in the case of textures. There are 250 classes in this dataset, and each class has 100 samples and 384 by 235 pixel pictures. It is useful for content-based image retrieval tasks. The ALOT dataset contains a large number of other semantic classifications, such as clothes, spices, leaves, cigarettes, sands, fruits, vegetables, stones, seashells, textiles, coins, seeds, embossed fabrics, bubbles, little repeating patterns, and more. These diverse classes provide the necessary textural details, various objects, shapes, and spatial properties needed for effective image classification. This extensive dataset assess the presented method's efficiency and adaptability. It demonstrates its ability to handle texture images with various features from the same semantic categories, such as large, complex, overlaid, textured, background, and foreground elements. The presented method uses color coefficients, multi-scale filters, Gaussian filters, L2 normalization stages, and other techniques to yield meaningful results for a range of textures in images. To accurately categorize images, multi-scale filtering is applied at varying scale spacing and levels to produce excellent average precision ratios for distinct texture images.

**Fig 16:**
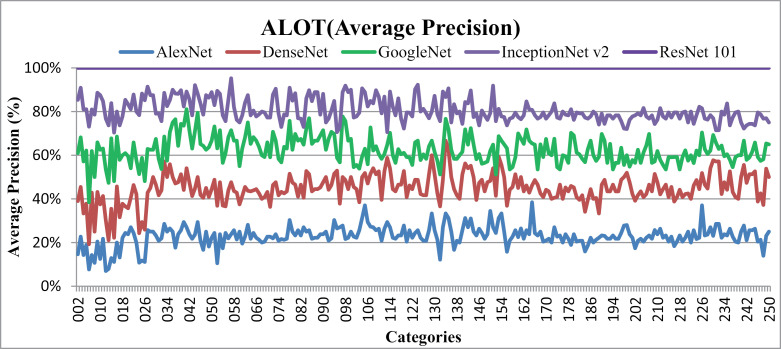
ALOT Database Average precision (%).

Fig 16 shows Average Precision rate (%) for each category of objects in ALOT database for multiple CNN architectures. Using ResNet-101, the presented approach obtains impressive AP values of up to 100% in most ALOT categories. Interestingly, the presented method performs better in a variety of image categories with different forms and colors. Images are successfully identified by combining RGB coefficients, spatial mapping, multi-scale filtering, and scale spacing with CNN features. Presented method is tested on many images with a variety of hues that belong to categories including fruits, vegetables, spices, and seeds. In particular, AP rates of more than 90% with InceptionNet v2, more than 80% with GoogLeNet, more than 60% with DenseNet and almost 40% with AlexNet are obtained for these image classes.

**Fig 17:**
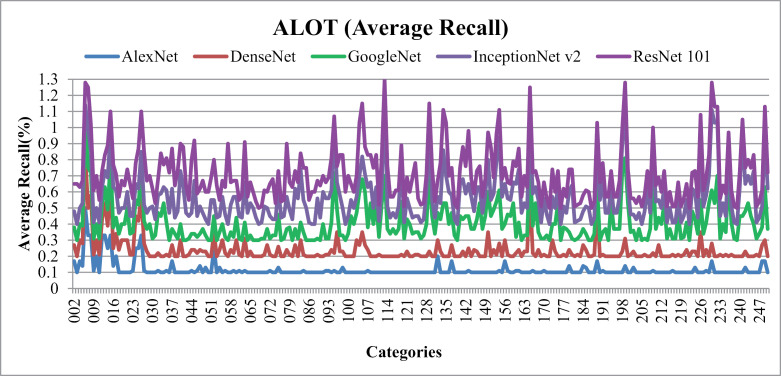
ALOT Database Average Recall (%).

Fig 17 depicts the Average Recall (%) for ALOT database categories. Applied to several ALOT picture groups, the presented method regularly achieves performance levels above 90% with ResNet-101 and above 80% with InceptionNet v2.Moreover, high accuracy is achieved for classification and exploration of semantic groupings comprising vertical lines, bubble textures, embossed textiles, horizontal lines, small repetitive patterns, and other visually unique features.

It is clear that the presented method performs exceptionally well in obtaining outcomes in a wide range of image categories, exhibiting clear color and shape differences. The presented method shows accurate and effective image classification by carefully applying thresholding, vectorization techniques, and combining color vectors with CNN deep features.

**Fig 18:**
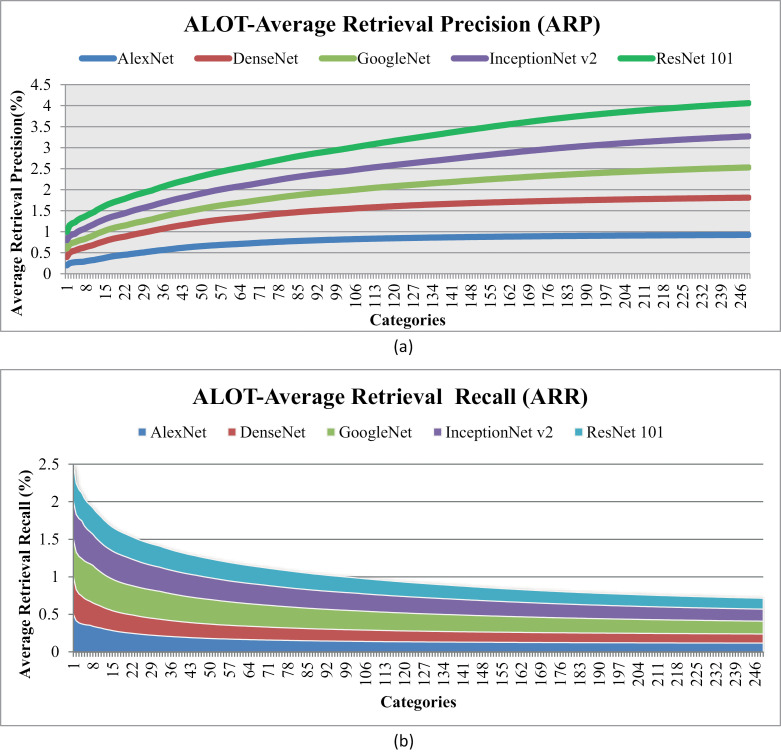
(a) ALOT Average Retrieval Precision (%). (b) ALOT Average Retrieval Recall (%).

Fig 18(a) shows Average Retrieval Precision (ARP) and Fig 18(b) shows Average Retrieval Recall (ARR) rates for ALOT over 250 categories. The results reveal that the majority of categories achieved ARP rates of over 85%, as depicted in Fig 18(a). However, one category showed a 70% ARP rate for the presented method. Fig 18(b) highlights the particularly high ARR rate observed for specific image categories. Results show exceptional mean average precision rates for the leaf, stone, and fabric categories, all achieving ARP rates ranging from 90% to 100%.Top of Form

**Fig 19:**
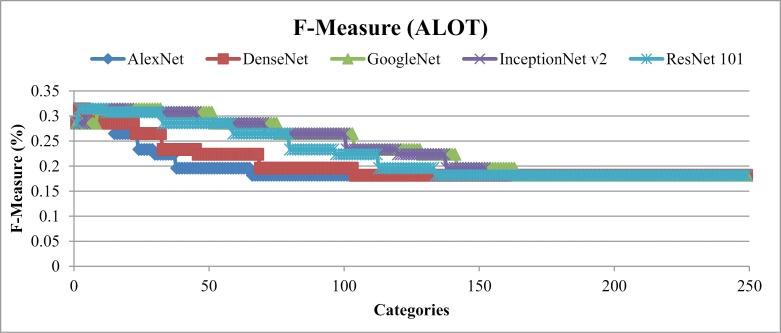
F-measure (ALOT).

**Fig 20:**
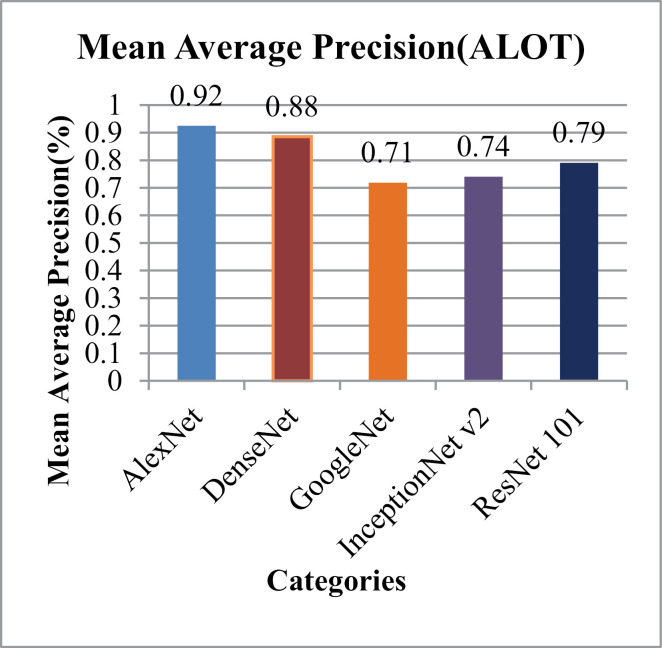
ALOT Mean Average Precision (%).

**Fig 21:**
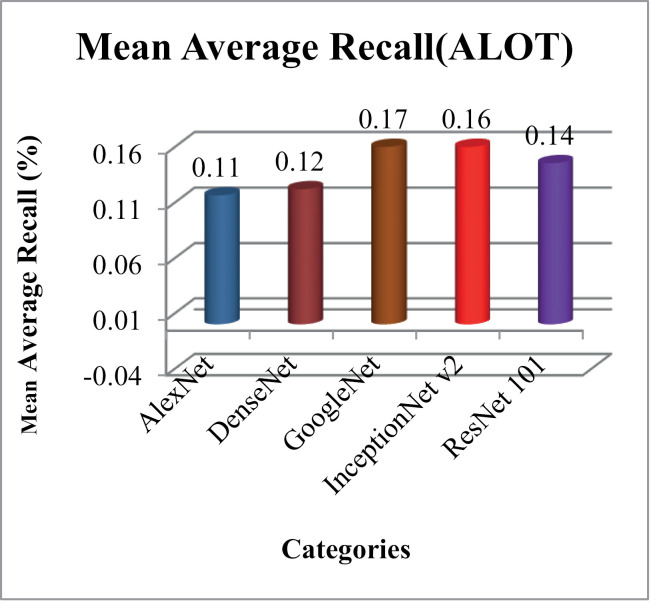
ALOT Mean Average Recall (%).

Fig 19 represents the F-measure results that range from 25% to 30% for all image categories. Fig 20 and 21 depict the mean Average Precision and mean Average Recall rates for ALOT categories. The presented method showed Highest mean Average Precision for AlexNet and almost 80% for the ResNet-101. InceptionNet v2 and GoogleNet attain highest mean Average Recall rates for the ALOT data set. It effectively categorize texture images within semantic groups that share similar features such as large, complex overlays, textures, and background and foreground objects. This is facilitated through the application of fixed-length filtering, recursive masking, color vectors, and L2 normalization steps. Model demonstrates significant capabilities in classifying images with diverse textures by utilizing CNN deep features in conjunction with Gaussian derivatives, thresholding, and corner detection techniques.

### 5.2. Experimentation on Tropical-Fruits

**Fig 22:**
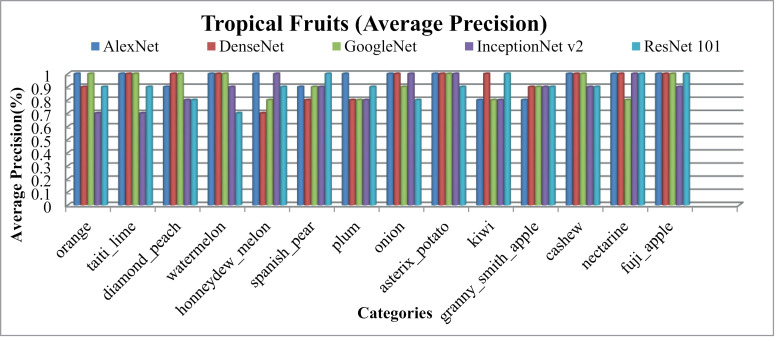
Tropical-Fruits Database Average Precision (%).

Fig 22 presents the notable performance metrics of the presented method on the Tropical Fruits database, including AP, AR, f-measure, and ARP, ARR, MAP and MAR rates. It's worth mentioning that presented approach achieves the highest results for most images within the Tropical Fruits categories, particularly those with similar shapes and colors. This success is attributed to the integration of spatial mapping, L2 normalization, color coefficients, and multilevel scaling with CNN features, enabling effective classification of tropical fruit images.

**Fig 23:**
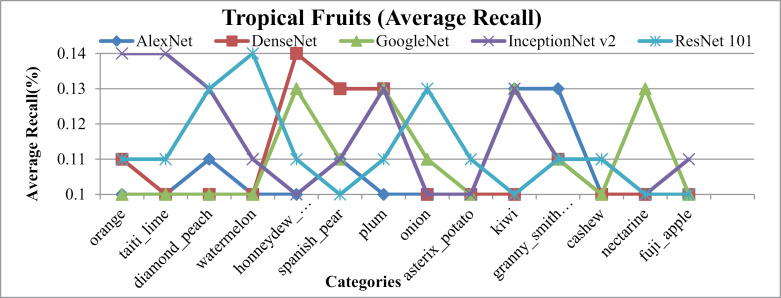
Tropical-Fruits Database Average Recall (%).

Fig 22 represents AP and Fig 23 show AR for Tropical Fruits database over various CNN benchmarks. Remarkably, presented method achieves exceptionally high rates, particularly with AlexNet, in 12 out of 15 tropical fruit classes. Additionally, it reports the highest AP rate when ResNet-101 for the Spanish Pear and Fuji-apple and Kiwi categories. In particular, AP rates of more than 95% with AlexNet, more than 90% with DenseNet and GoogleNet, ResNET-101 and almost 85% with InceptionNet v2 are obtained for these image classes. Relative result values are also presented in the Table 1.

**Table 1:** The Average Precision, Average Recall Result for Tropical-Fruits.

Tropical-Fruits (AP,AR Results)										
Category	AlexNet		DenseNet		GoogleNet		InceptionNet v2		ResNet-101	
orange	1	0.1	0.9	0.11	1	0.1	0.7	0.14	0.9	0.11
taiti_lime	1	0.1	1	0.1	1	0.1	0.7	0.14	0.9	0.11
diamond_peach	0.9	0.11	1	0.1	1	0.1	0.8	0.13	0.8	0.13
watermelon	1	0.1	1	0.1	1	0.1	0.9	0.11	0.7	0.14
honneydew_melon	1	0.1	0.7	0.14	0.8	0.13	1	0.1	0.9	0.11
spanish_pear	0.9	0.11	0.8	0.13	0.9	0.11	0.9	0.11	1	0.1
plum	1	0.1	0.8	0.13	0.8	0.13	0.8	0.13	0.9	0.11
onion	1	0.1	1	0.1	0.9	0.11	1	0.1	0.8	0.13
asterix_potato	1	0.1	1	0.1	1	0.1	1	0.1	0.9	0.11
kiwi	0.8	0.13	1	0.1	0.8	0.13	0.8	0.13	1	0.1
granny_smith_apple	0.8	0.13	0.9	0.11	0.9	0.11	0.9	0.11	0.9	0.11
cashew	1	0.1	1	0.1	1	0.1	0.9	0.11	0.9	0.11
nectarine	1	0.1	1	0.1	0.8	0.13	1	0.1	1	0.1
fuji_apple	1	0.1	1	0.1	1	0.1	0.9	0.11	1	0.1

**Fig 24:**
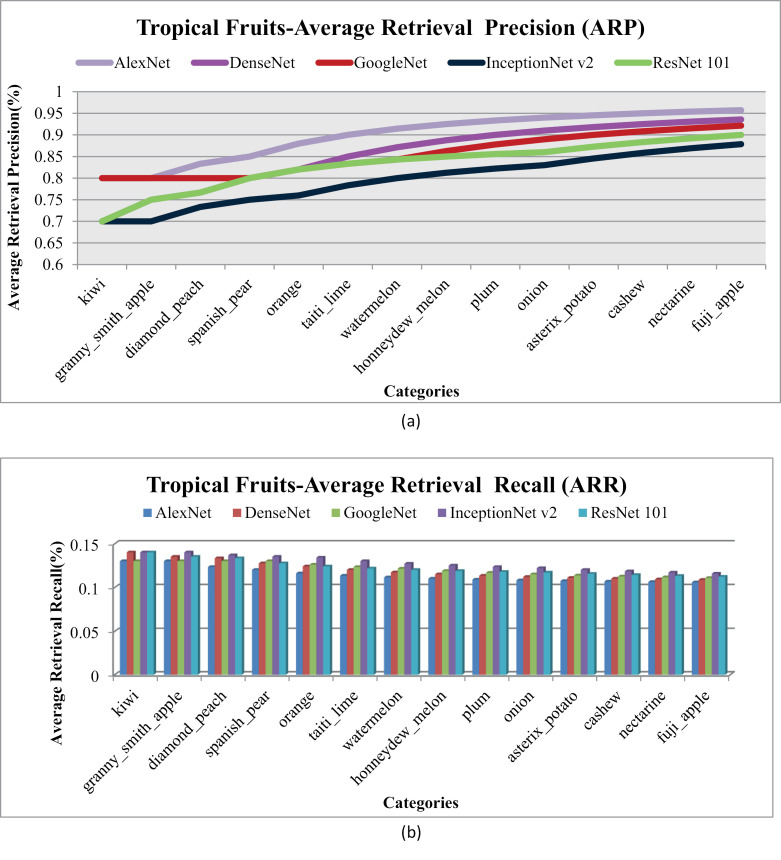
(a) Tropical-Fruits Average Retrieval Precision (%). (b). Tropical-Fruits Average Retrieval Recall (%).

Fig 24(a) represents ARP and Fig 24(b) represents the rates for ARR for Tropical Fruits database. Significant ARP rates particularly with AlexNet and DenseNet, for most categories, showcasing the robustness of presented method for the tropical fruits dataset.

**Fig 25:**
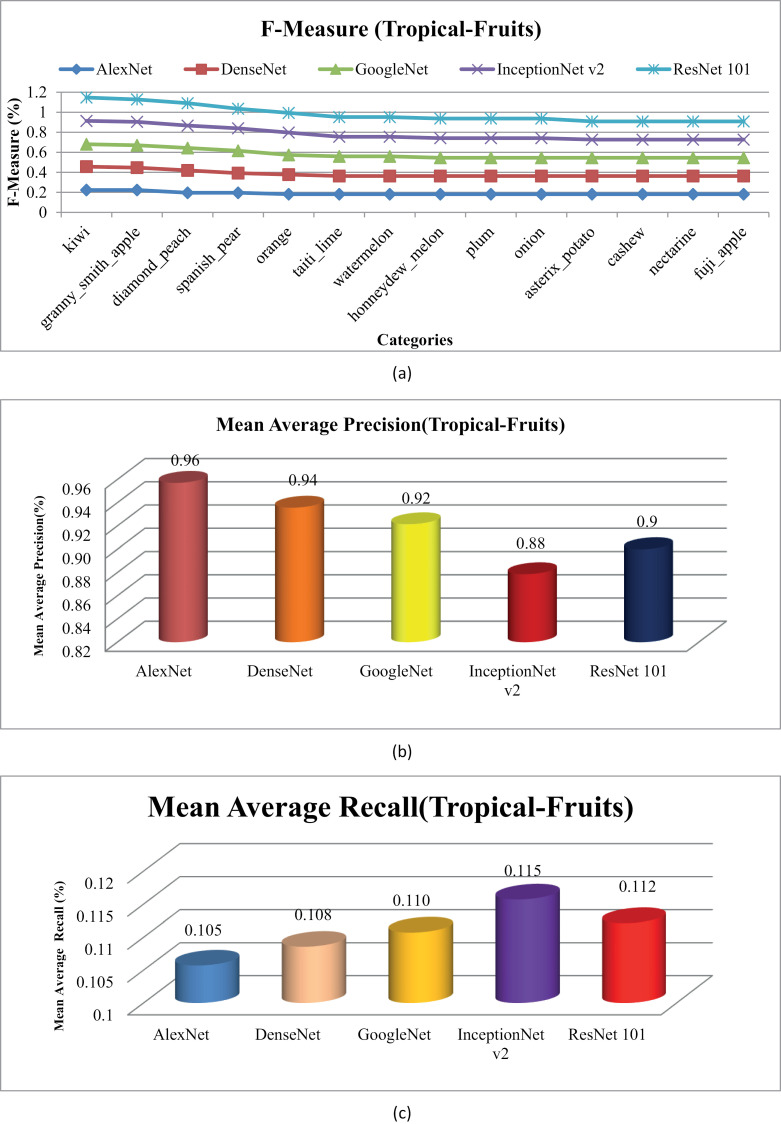
(a). Tropical-Fruits F-measure. (b). Tropical-Fruits MAP. (c). Tropical-Fruits MAR.

Fig 25(a) depicts the F-measure results for the Tropical Fruits database. Results demonstrate presented approach exhibits outstanding performance across the Tropical Fruits database, particularly with AlexNet, DenseNet and GoogLeNet. MAP and MAR rates using various CNN are shown in Fig 25(b) and 25(c). It demonstrates a remarkable MAP of 95% using AlexNet, 93% using DenseNet and 92% using GoogLeNet and 90% using AlexNet for the tropical fruits dataset.

### 5.3. Experimentation on Cifar-10

**Fig 26:**
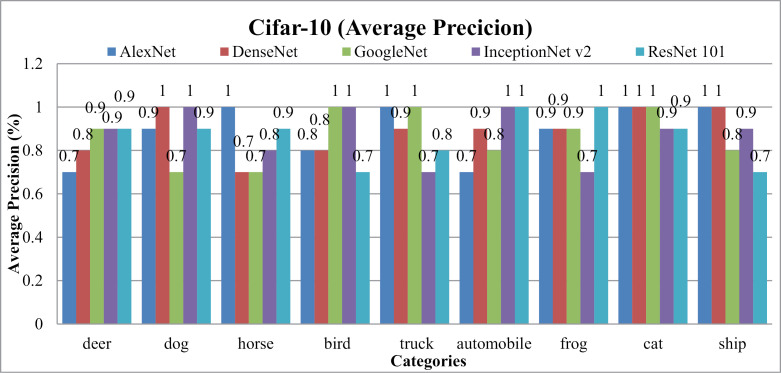
Cifar-10 Database Average Precision (%).

**Fig 27:**
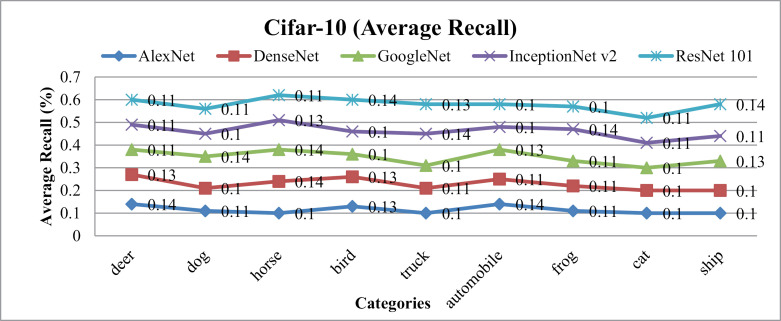
Cifar-10 Database Average Recall (%).

In Figs 26 and 27, the presented method's AP and AR performance is shown for the Cifar-10 dataset using AlexNet, DenseNet, and GoogleNet, InceptionNetv2 and ResNet-101. AlexNet and DenseNet show the highest AP ratios across most categories within the Cifar-10 dataset. The presented method achieves AP rates above 85% with AlexNet and DenseNet and no less than 75% with InceptionNet v2 across various semantic groups in the dataset. This approach excels, especially in scenarios involving small, simulated backgrounds with multiple objects, owing to its strong object recognition capability. Additionally, the presented method achieves remarkable AP ratios in classifying images with cluttered, complex, and overlapping objects.

**Fig 28:**
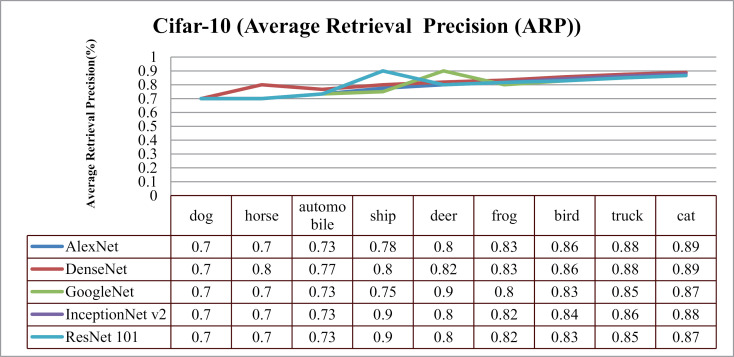
Cifar-10 Database Average Retrieval Precision (%).

**Fig 29:**
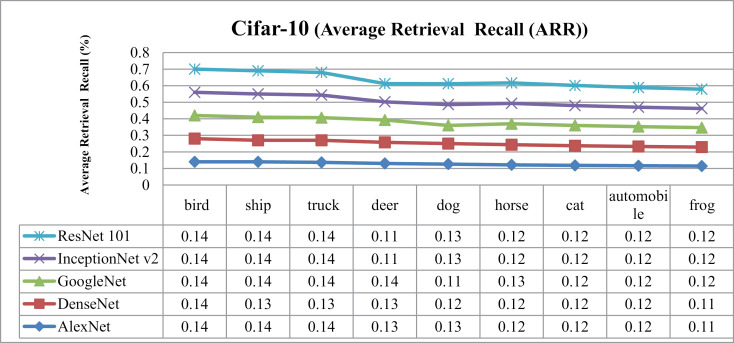
Cifar-10 Database Average Retrieval Recall (%).

In the context of the Cifar-10 database, presented approach delivers remarkable ARP ratios, particularly using AlexNet and DenseNet for categories like airplanes, dogs, frogs, ships, and birds, as evidenced in Fig 28. Moreover, in Fig 29, across other categories, method achieves consistent performance with above 85% average retrieval recall ratios highlighting its effectiveness within the dataset. The results demonstrate improved ARP rates for DenseNet and AlexNet employed extracted features.

**Fig 30:**
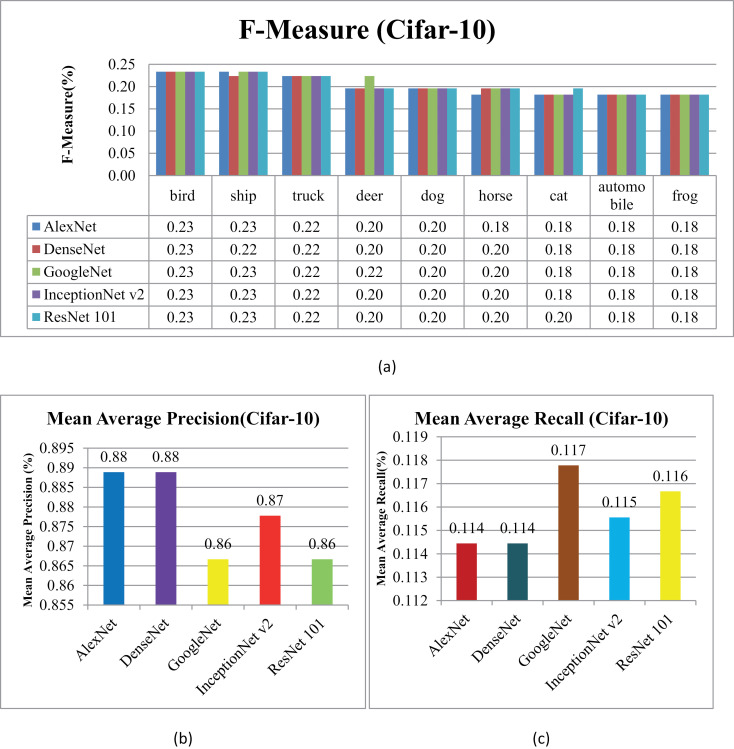
(a) Cifar-10 Database F-Measure. (b) Cifar-10 Database Mean Average Precision (%). (c) Cifar-10 Database Mean Average Recall (%).

In Fig 30(a), the presented approach reports outstanding f-measure ratios for images characterized by large, mimicked, and complex foreground and background elements. The method demonstrates significant f-measure ratios across a range of scenarios when utilizing DenseNet, AlexNet, GoogLeNet, InceptionNet, and ResNet-101 architectures.

Specifically, in Fig 30(b) the presented approach achieves a mean average precision of more than 85% with AlexNet and DenseNet and InceptionNet v2 for the Cifar-10 dataset. Additionally, presented method delivers competitive performance with AlexNet and DenseNet, achieving notable F-measure ratios in various image contexts.

Fig 30(c) shows the mean average Recall ratio for the Cifar-10. The model achieves MAR values of 0.11 with AlexNet and DenseNet, 0.115 with InceptionNet v2 and, and 0.117 with GoogleNet on the Cifar-100 dataset. Presented method demonstrates equal proficiency in handling dataset harmonization, regardless of image size, effectively addressing both large and small images. Moreover, it excels in processing complex images with intertwined foreground and background content, ensuring accurate interpretation even in scenarios where objects are indistinct or overlapping.

### 5.4. Experimentation on Corel-10k

**Fig 31:**
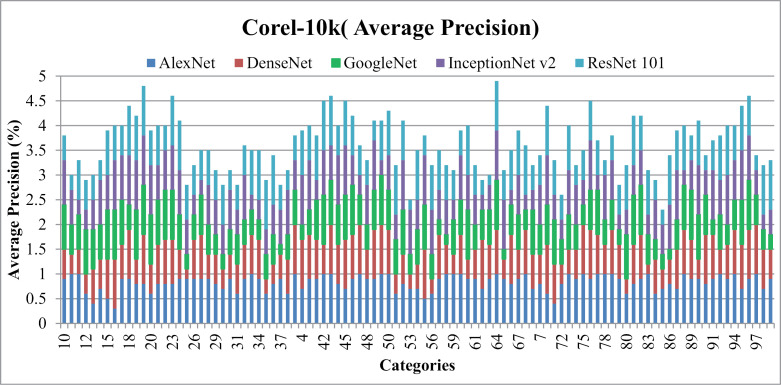
Corel-10k Database Average Precision (%).

Fig 31 presents average precision results for Corel-10k. It gives more than 85% accuracy using AlexNet, and 70% with the DenseNet, AlexNet and InceptionNet. It effectively categorizes images from diverse groups, including various foregrounds and backgrounds, complex blobs, overlays, and cluttered scenes. This is achieved through the use of color coefficients, distance measures, L1 and L2 norms, color signatures, architectural bonding, signature influencing, dataset harmonization, thresholding, factoring, and regioning techniques.

**Fig 32:**
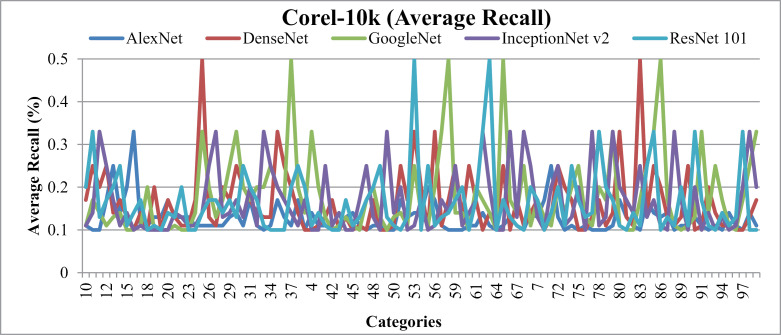
Corel-10k Database Average Recall (%).

Fig 32 shows the average Recall results for Corel-10k over AlexNet, DenseNet, InceptionNet v2, and GoogLeNet and ResNet-101 architectures. Significantly, presented approach achieves outstanding average Recall across most classes within Corel-10k, demonstrating dominance due to its incorporation of color channeling, auto-correlation, L2 normalization, shape parameters, and straddling.

Table 2 shows the ARP and ARP rates for the Corel-10k. Results show that AlexNet gives 90% ARP, DenseNet and Inception 80% ARP for the categories of Corel-10k. The results also show above 0.11 ARR results with AlexNet, above 0.2 ARR ratios by Dense Net, 0.25 ARR results with InceptionNet and above 0.3 using ResNet-101 for Corel-10k. The presented method here operates on color coefficients by applying suitable displacements at L1 and L2 levels. However, it does not address the interpretability aspects of feature sets, as it only selects the heads connected to the signature-influencing dimension.

**Table 2:** The ARP, ARR Results for Corel-10k.

Corel-10k (ARP,ARR Results)										
Category	AlexNet		DenseNet		GoogleNet		InceptionNet v2		ResNet-101	
2	0.73	0.14	0.80	0.15	0.53	0.25	0.87	0.12	0.90	0.11
3	0.70	0.14	0.70	0.18	0.30	0.33	0.80	0.14	0.85	0.12
4	0.70	0.14	1.00	0.10	0.30	0.33	1.00	0.10	0.90	0.11
5	0.85	0.12	1.00	0.10	0.65	0.22	0.65	0.22	0.85	0.12
6	0.90	0.11	0.93	0.11	0.67	0.19	0.73	0.18	0.73	0.15
7	0.88	0.12	0.85	0.13	0.65	0.19	0.75	0.17	0.70	0.15
8	0.88	0.12	0.82	0.13	0.58	0.21	0.66	0.20	0.68	0.16
9	0.88	0.12	0.75	0.15	0.63	0.20	0.72	0.18	0.72	0.15
10	0.90	0.11	0.60	0.17	0.90	0.11	0.90	0.11	0.50	0.20
11	0.90	0.11	0.70	0.16	0.68	0.18	0.69	0.19	0.70	0.15
12	0.87	0.12	0.67	0.17	0.70	0.17	0.66	0.20	0.69	0.15
13	0.82	0.13	0.67	0.17	0.71	0.17	0.65	0.20	0.67	0.16
14	0.81	0.13	0.66	0.17	0.71	0.16	0.67	0.19	0.65	0.17
15	0.78	0.14	0.68	0.16	0.73	0.16	0.68	0.18	0.67	0.16
16	0.75	0.15	0.70	0.16	0.75	0.15	0.70	0.18	0.67	0.16
17	0.76	0.15	0.70	0.16	0.76	0.15	0.71	0.17	0.66	0.16
18	0.77	0.15	0.72	0.15	0.75	0.15	0.73	0.17	0.69	0.16
19	0.77	0.15	0.71	0.16	0.76	0.15	0.75	0.16	0.70	0.15
20	0.76	0.15	0.70	0.16	0.78	0.15	0.76	0.16	0.70	0.15
21	0.76	0.15	0.71	0.16	0.78	0.15	0.76	0.16	0.71	0.15
22	0.76	0.15	0.72	0.15	0.79	0.14	0.76	0.16	0.69	0.15
23	0.77	0.15	0.73	0.15	0.81	0.14	0.77	0.16	0.71	0.15
24	0.77	0.14	0.72	0.15	0.80	0.14	0.78	0.15	0.72	0.15
25	0.78	0.14	0.70	0.17	0.78	0.15	0.77	0.15	0.72	0.15
26	0.78	0.14	0.70	0.17	0.77	0.15	0.76	0.16	0.72	0.15
27	0.79	0.14	0.71	0.16	0.77	0.15	0.74	0.16	0.71	0.15
28	0.79	0.14	0.70	0.16	0.76	0.15	0.74	0.16	0.71	0.15
29	0.79	0.14	0.70	0.17	0.75	0.16	0.74	0.16	0.71	0.15
30	0.80	0.14	0.69	0.17	0.74	0.16	0.74	0.16	0.70	0.15
31	0.79	0.14	0.69	0.17	0.73	0.16	0.73	0.16	0.69	0.16
32	0.79	0.14	0.69	0.17	0.72	0.16	0.74	0.16	0.69	0.16
33	0.80	0.14	0.69	0.16	0.72	0.16	0.72	0.17	0.69	0.16
34	0.80	0.13	0.69	0.16	0.71	0.16	0.71	0.17	0.70	0.15
35	0.80	0.14	0.68	0.17	0.70	0.16	0.71	0.17	0.71	0.15
36	0.80	0.14	0.67	0.17	0.70	0.16	0.70	0.17	0.72	0.15
37	0.80	0.14	0.67	0.17	0.68	0.17	0.70	0.17	0.71	0.15
38	0.79	0.14	0.67	0.17	0.68	0.18	0.71	0.17	0.71	0.15
39	0.80	0.14	0.68	0.17	0.68	0.17	0.71	0.17	0.70	0.16
40	0.80	0.13	0.68	0.17	0.67	0.18	0.71	0.17	0.70	0.16
41	0.81	0.13	0.69	0.17	0.68	0.17	0.71	0.17	0.71	0.15
42	0.81	0.13	0.68	0.17	0.68	0.17	0.71	0.17	0.71	0.15
43	0.82	0.13	0.69	0.16	0.69	0.17	0.71	0.17	0.72	0.15
44	0.81	0.13	0.70	0.16	0.69	0.17	0.72	0.16	0.72	0.15
45	0.81	0.13	0.70	0.16	0.70	0.17	0.72	0.16	0.72	0.15
46	0.81	0.13	0.71	0.16	0.70	0.17	0.72	0.16	0.72	0.15
47	0.82	0.13	0.71	0.16	0.70	0.17	0.71	0.16	0.72	0.15
48	0.82	0.13	0.71	0.16	0.70	0.17	0.72	0.16	0.72	0.15
49	0.82	0.13	0.72	0.16	0.70	0.17	0.72	0.16	0.71	0.15
50	0.83	0.13	0.72	0.16	0.70	0.17	0.72	0.16	0.71	0.15
51	0.82	0.13	0.71	0.16	0.70	0.17	0.72	0.16	0.72	0.15
52	0.82	0.13	0.71	0.16	0.71	0.16	0.72	0.16	0.72	0.15
53	0.82	0.13	0.70	0.16	0.70	0.17	0.73	0.16	0.71	0.16
54	0.82	0.13	0.70	0.16	0.70	0.17	0.72	0.16	0.72	0.16
55	0.81	0.13	0.71	0.16	0.70	0.16	0.73	0.16	0.71	0.16
56	0.81	0.13	0.70	0.17	0.70	0.16	0.73	0.16	0.71	0.16
57	0.81	0.13	0.70	0.16	0.69	0.17	0.73	0.16	0.71	0.16
58	0.81	0.13	0.70	0.16	0.68	0.17	0.73	0.16	0.71	0.16
59	0.81	0.13	0.69	0.17	0.68	0.17	0.72	0.16	0.71	0.16
60	0.82	0.13	0.69	0.17	0.69	0.17	0.72	0.16	0.72	0.16
61	0.82	0.13	0.69	0.17	0.69	0.17	0.72	0.16	0.72	0.16
62	0.82	0.13	0.69	0.17	0.68	0.17	0.71	0.16	0.71	0.16
63	0.82	0.13	0.69	0.17	0.69	0.17	0.71	0.16	0.70	0.17
64	0.82	0.13	0.70	0.17	0.69	0.17	0.71	0.16	0.70	0.16
65	0.82	0.13	0.69	0.17	0.68	0.18	0.72	0.16	0.70	0.16
66	0.82	0.13	0.70	0.17	0.68	0.18	0.71	0.16	0.70	0.16
67	0.82	0.13	0.70	0.17	0.68	0.18	0.71	0.16	0.71	0.16
68	0.82	0.13	0.70	0.16	0.68	0.18	0.71	0.17	0.71	0.16
69	0.82	0.13	0.70	0.16	0.68	0.18	0.70	0.17	0.71	0.16
70	0.82	0.13	0.70	0.16	0.68	0.17	0.71	0.17	0.71	0.16
71	0.81	0.13	0.70	0.16	0.69	0.17	0.70	0.17	0.71	0.16
72	0.81	0.13	0.70	0.16	0.68	0.17	0.70	0.17	0.71	0.16
73	0.82	0.13	0.70	0.16	0.68	0.17	0.70	0.17	0.71	0.16
74	0.82	0.13	0.70	0.16	0.68	0.17	0.70	0.17	0.71	0.16
75	0.82	0.13	0.70	0.16	0.68	0.18	0.70	0.17	0.71	0.16
76	0.82	0.13	0.70	0.16	0.68	0.17	0.71	0.17	0.71	0.16
77	0.82	0.13	0.71	0.16	0.68	0.17	0.70	0.17	0.71	0.16
78	0.83	0.13	0.70	0.16	0.68	0.17	0.70	0.17	0.70	0.16
79	0.83	0.13	0.71	0.16	0.68	0.17	0.70	0.17	0.70	0.16
80	0.82	0.13	0.70	0.16	0.68	0.17	0.70	0.17	0.70	0.16
81	0.82	0.13	0.70	0.16	0.68	0.17	0.70	0.17	0.71	0.16
82	0.83	0.13	0.71	0.16	0.69	0.17	0.70	0.17	0.71	0.16
83	0.83	0.13	0.70	0.17	0.69	0.17	0.70	0.17	0.71	0.16
84	0.82	0.13	0.70	0.17	0.68	0.17	0.70	0.17	0.70	0.16
85	0.82	0.13	0.70	0.17	0.68	0.17	0.70	0.17	0.70	0.17
86	0.82	0.13	0.69	0.17	0.67	0.18	0.70	0.17	0.70	0.16
87	0.82	0.13	0.69	0.17	0.67	0.18	0.70	0.17	0.70	0.16
88	0.82	0.13	0.70	0.17	0.68	0.18	0.70	0.17	0.71	0.16
89	0.82	0.13	0.70	0.17	0.68	0.18	0.70	0.17	0.70	0.16
90	0.82	0.13	0.70	0.17	0.68	0.18	0.69	0.17	0.70	0.17
91	0.83	0.13	0.70	0.17	0.68	0.18	0.70	0.17	0.70	0.17
92	0.83	0.13	0.70	0.17	0.68	0.18	0.70	0.17	0.70	0.17
93	0.83	0.13	0.70	0.17	0.67	0.18	0.70	0.17	0.70	0.16
94	0.83	0.13	0.70	0.16	0.67	0.18	0.70	0.17	0.70	0.16
95	0.83	0.13	0.71	0.16	0.68	0.18	0.71	0.17	0.71	0.16
96	0.83	0.13	0.71	0.16	0.68	0.18	0.71	0.16	0.71	0.16
97	0.83	0.13	0.71	0.16	0.68	0.18	0.71	0.17	0.70	0.17
98	0.83	0.13	0.71	0.16	0.67	0.18	0.70	0.17	0.71	0.16
99	0.83	0.13	0.71	0.16	0.67	0.18	0.70	0.17	0.71	0.16
100	0.83	0.13	0.71	0.16	0.67	0.18	0.70	0.17	0.71	0.17

**Fig 33:**
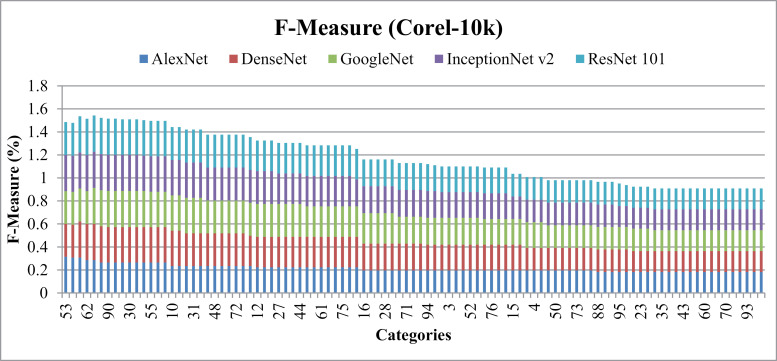
Corel-10k F-measure.

Fig 33 reports f-measure ratios Corel-10k dataset images with varying shapes and textures that share common patterns, shapes, and colors, while other image groups encompass diverse object patterns.

**Fig 34:**
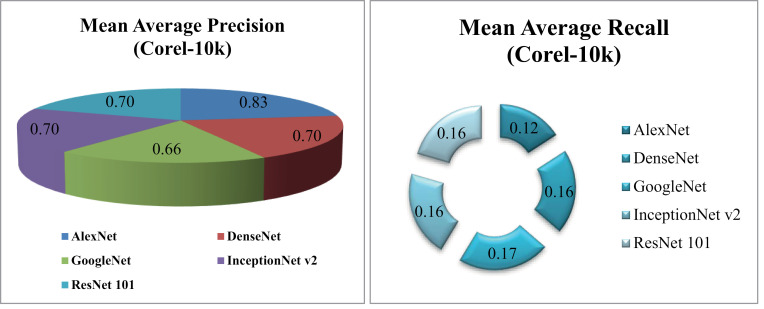
(a).Corel-10k Mean Average Precision (%). (b).Corel-10k Mean Average Recall (%).

Results show83% MAP using AlexNet, 70% MAP with DenseNet, ResNet-101 and InceptionNet v2 and more than 60%with GoogleNet on the Corel-10k dataset in Fig 34 (a). Additionally, the MAR graph in Fig 34 (b) illustrates values of 0.12 MAR for AlexNet, 0.16 MAR for DenseNet, InceptionNet v2, andResNet-101, and 0.17 MAR for GoogleNet on the same dataset.

### 5.5. Experimentation on Zubud

**Fig 35:**
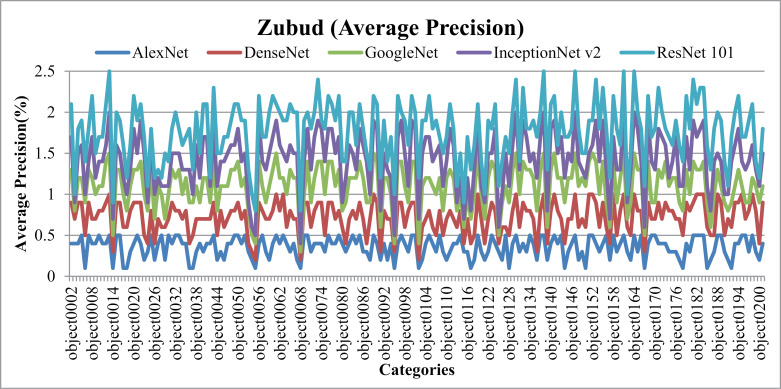
Zubud Database Average Precision (%).

**Fig 36:**
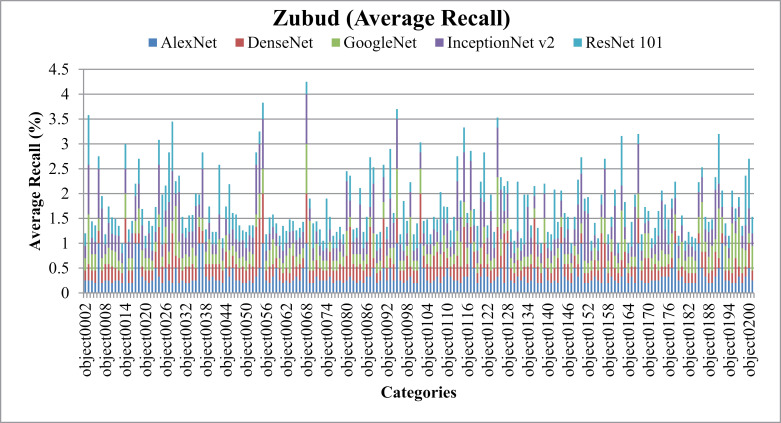
Zubud Database Average Recall (%).

Fig 35 illustrates notable AP and Fig 36 shows AR outcomes achieved with the Zubud dataset. It’s observed that the presented approach demonstrates enhanced AP performance across various image classes within the dataset, characterized by distinct colors, textures, and shapes. Notably, the presented approach attains AP rates surpassing 90% with DenseNet across the majority of image groups in Zubud. Additionally, results indicate AP rates exceeding 85% with AlexNet, over 80% with ResNet-101, and above 77% with GoogleNet across most semantic groups within Zubud.

**Fig 37:**
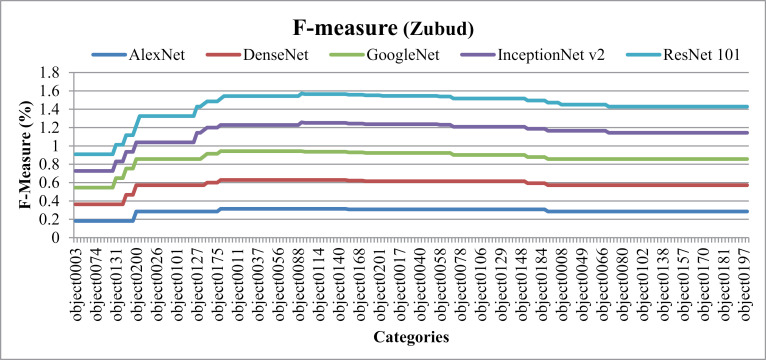
Zubud Database F-Measure.

In Fig 37, the presented model showcases the f-measure results obtained across various scenarios within the Zubud dataset. Specifically, when utilizing DenseNet, the model achieves f-measure ratios ranging from 25% to 30%. Similarly, with ResNet101, f-measure ratios fall within the range of 18% to 24%, while GoogLeNet yields ratios of 19% to 24%. Additionally, when employing AlexNet, the f-measure rates range from 19% to 27%. These results are indicative of the model's performance in detecting and classifying large, cluttered, overlaid, complex, and color-dominant objects present within the Zubud dataset.

**Fig 38:**
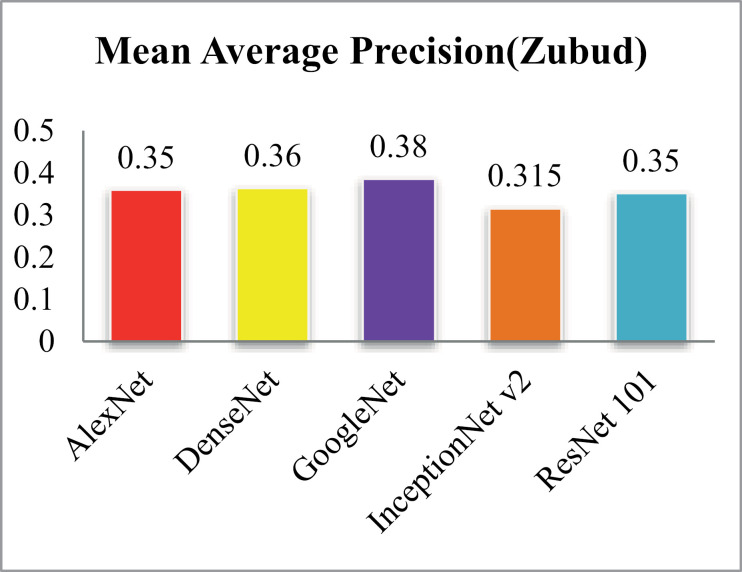
Zubud Database Mean Average Precision (%).

**Fig 39:**
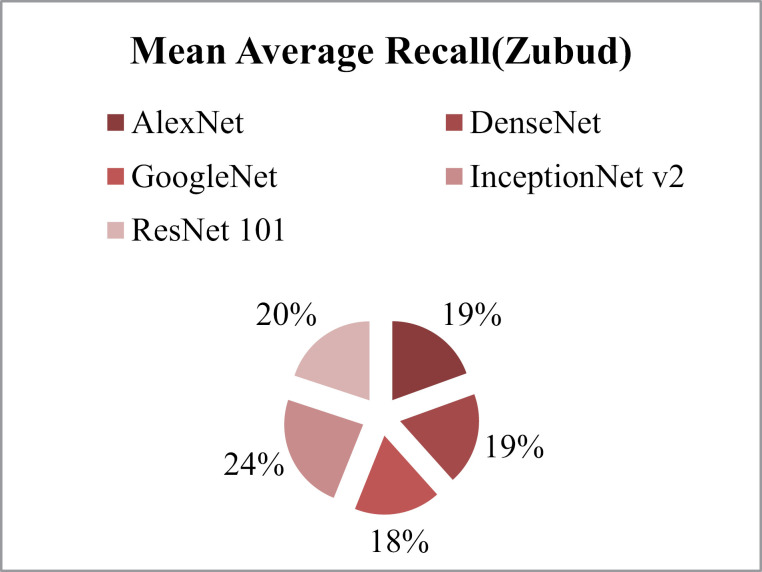
Zubud Database Mean Average Recall (%).

MAP is shown in Fig 38 and MAR results are shown in Fig 39.Results highlight that the method shows 83% ARP using GoogleNet, 75% ARP with DenseNet, AlexNet and AlexNet 101 and more than 70% with InceptionNet v2 on the Zubud dataset.

The presented approach includes innovative feature fusion algorithms to provide combined and larger features sets. Information loss during merging may cause increase in experimental costs. The presented methodology minimizes computational costs by ensuring the stringent signature lengths. This research is significant since it integrates various complicated CNN architectures that have not been explored collectively in earlier research. Adverse architectural bonding along with multiple datasets is formed. The presented method tested on multiple datasets provides high precision rates for deep image sensing.

## 6. Discussion

In this paper, a novel concept for deep image features sensing with multilevel fusion within CNN architectures and particularly towards the cross domain benchmarks is presented. CNNs have emerged as a core component of image processing because of abilities to automatically learn spatial architectures, but there are several constraints about employing CNNs for cross-domain tasks and variability of their applicable data types. To differentiate the presented work from existing CNN-based studies, the focus is on three primary areas of innovation. 1) In general, most of the existing CNN architectures utilize pooling initializing and feature extracting functions within single-domain layers, which hinders their potential to incorporate data from different levels of abstraction. However, presented method provides a multilevel fusion mechanism which allows the model to accommodate for merging features from different layers by providing cross-layer integration thus allowing the model to capture details that would have been missing in other approaches. This multi-level fusion increases the model's capability to work in various tasks where representation at both the aggregate and detailed levels is required. 2) Most of the existing work on CNN architecture has been done in terms of its performance in a particular area which severely restricts the permutation of the CNN model while facing two different data sources or domains. The benchmarking approach employed in presented method combines a cross-domain methodology to evaluate the CNN robustness across multiple imaging types, which helps to fill a crucial research gap that overlooks the CNN applicability to real-world multi-domain imaging. This is especially useful when the model needs to be adaptive since it increases the flexibility of CNNs in the cross-domain strategy from the conventional applications by domain. Besides cross domain benchmarking, it advances CNN to filter out more subtle features by a method of personalized feature sensing. 3) Unlike the traditional CNNs, that could ignore lots of differences, the presented model is specifically designed to detect and differentiate fine-grained patterns even in complex, cross-domain images. This is achieved by adjusting Convolutional layers to focus on detailed feature extraction, a method that offers substantial improvements for applications such as medical imaging and other domains where small, complex details are critical. These methods are implemented in the presented CNN framework, which addresses various limitations of the contemporary CNN research by offering a more flexible and effective model for image analysis. Thus, this paper provides important findings for further development of CNN methods, especially as it pertains to the introduced multilevel feature fusion and the examination of cross-domain performance on more complex and realistic problem settings.

## 7. Conclusion

Accurate and efficient image retrieval has been vital in the digital age. This research has presented a CRIB-based deep image sensing technique with multi-level fusion on CNNs. The presented method has provided image identification and categorization based on a fusion of image feature descriptors with deep-learned features using CNNs to address image retrieval challenges across various semantic categories and datasets. Effective image retrieval and classification has been achieved through primitive feature vectors. Deep image sensing and synthesis have provided efficient detection of cluttered, overlaid, foreground, and background images. Image classification has been achieved through descriptor formation and matching, factorization, thresholding, and Gaussian filtering. Experimentation on standard benchmarks of Cifar-10, Tropical Fruits, ALOT, Corel-10k, and Zubud has validated the superiority of the presented method. Results have concluded that the presented method has correctly classified and retrieved images from various complex datasets and has provided high precision and recall rates for complex image categories. Fusion of conventional feature extraction methods to multilevel CNNs has improved the accuracy of image sensing and retrieval. Future extensions to the presented approach have aimed to make it pertinent to big data and deep cloud-based architectures.

## Supporting information

S1 File The file contains categories used for Data Availability Statements and their relevant definitions. (PDF)
