# Supplementary material for: Deep image features sensing with multilevel fusion for complex convolution neural networks & cross domain benchmarks
Source: PLoS One. 2025 Mar 18;20(3):e0317863. doi: 10.1371/journal.pone.0317863 (PMC11918433; doi:10.1371/journal.pone.0317863)
Supplement: S1 File — (PDF) [file pone.0317863.s001.pdf]

Supporting Information for:

Deep Image Features Sensing with Multilevel Fusion for Complex Convolution Neural Networks& Cross Domain Benchmarks

Contents:

No.	Topic	Page Number
1	Supplementary Figures	2
2	Supplementary Tables	8
3	Additional Methods and Details	9
4	Datasets and Benchmarks Information	9
5	Code and Reproducibility	10
6	References	11

1. Supplementary Figures

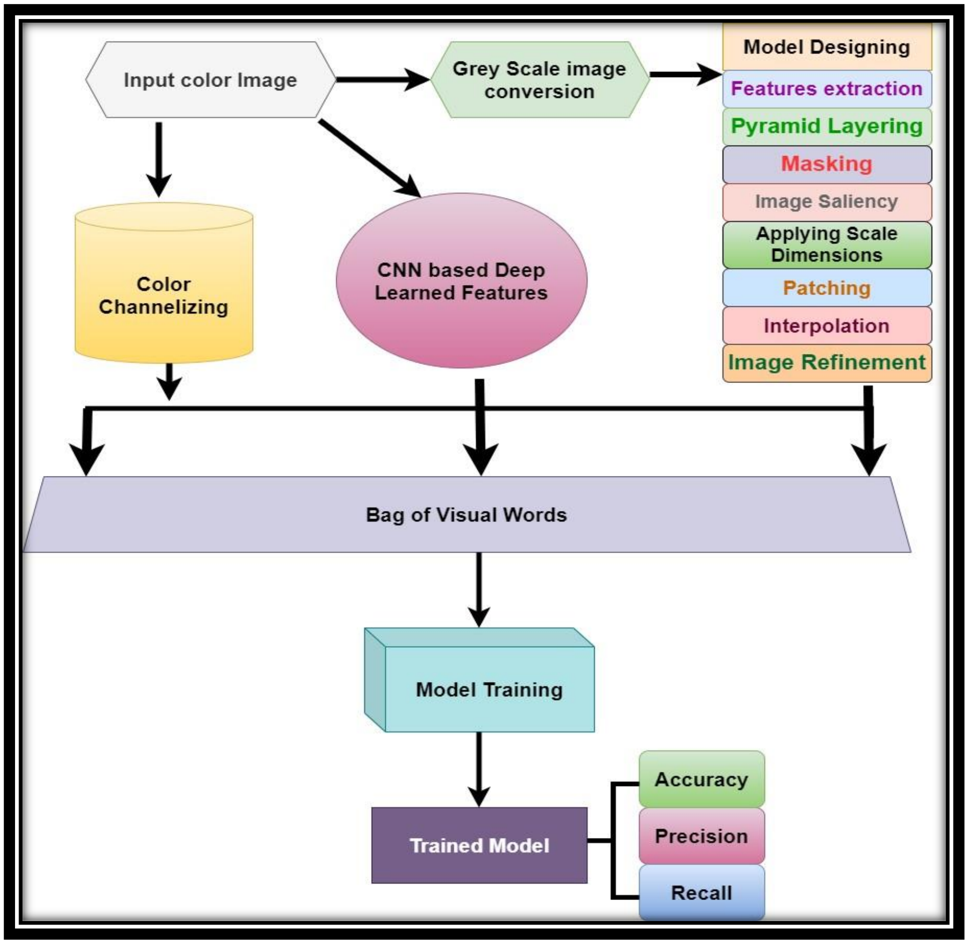

S1 Fig. Workflow of the Proposed System

These general details about the proposed image retrieval framework are summarized in S1 Fig. This pipeline consists of interest point detection, color, shape and object-based feature extraction, BoVW, and fusion of extracted features with deep features gained from a CNN.

Feature Distributions for Sample Datasets:

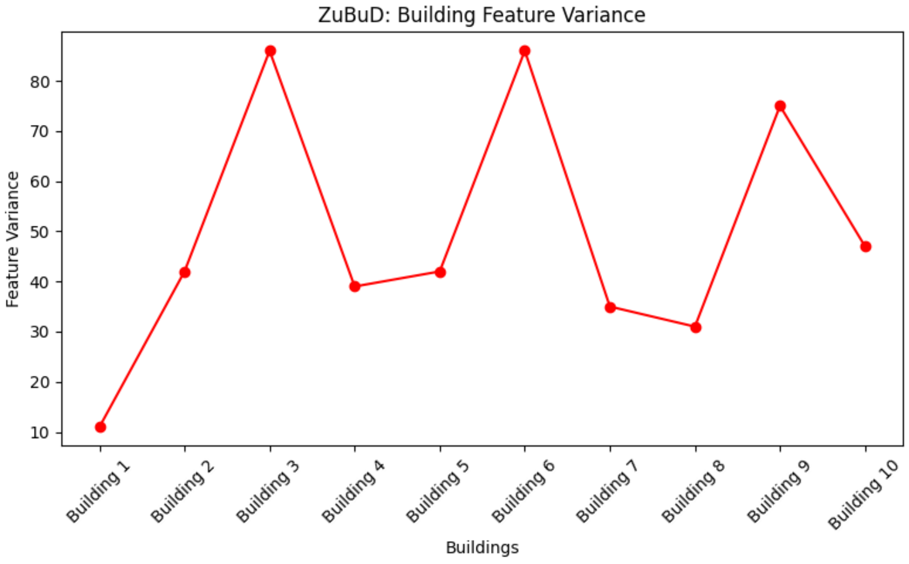

S2 Fig. Feature Distributions for Zubud Dataset

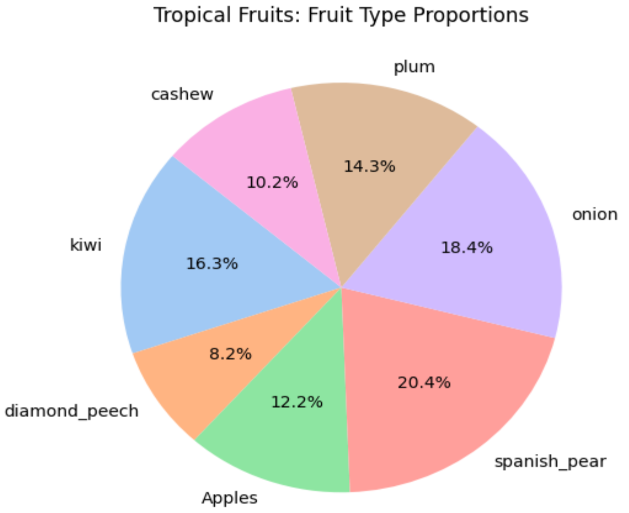

S3 Fig. Feature Distributions for Tropical-Fruits Dataset

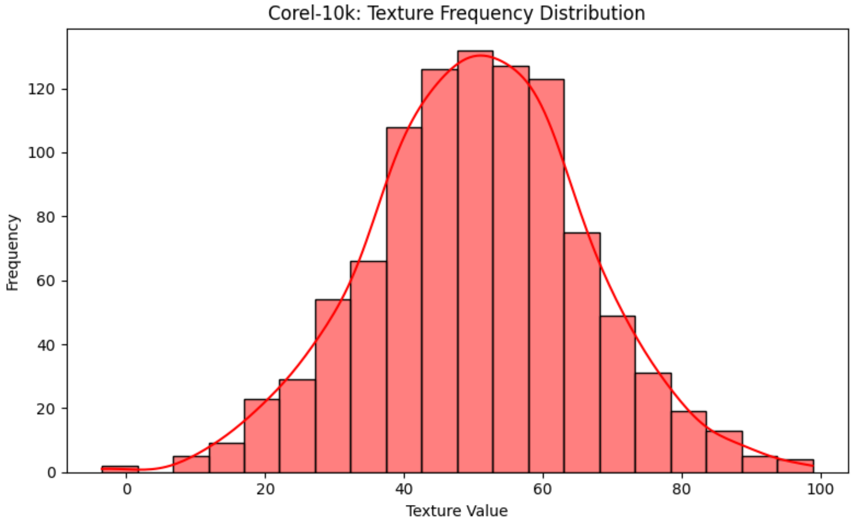

S4 Fig. Feature Distributions for Corel-10k Dataset

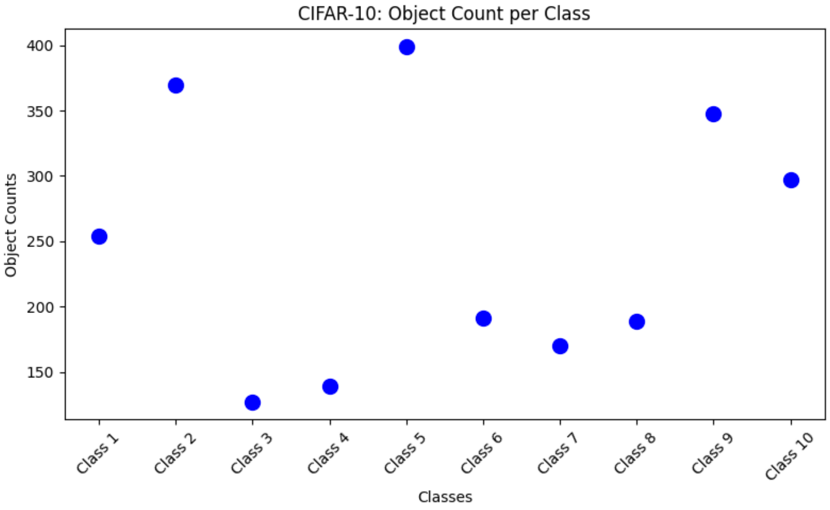

S5 Fig. Feature Distributions for Cifar-10 Dataset

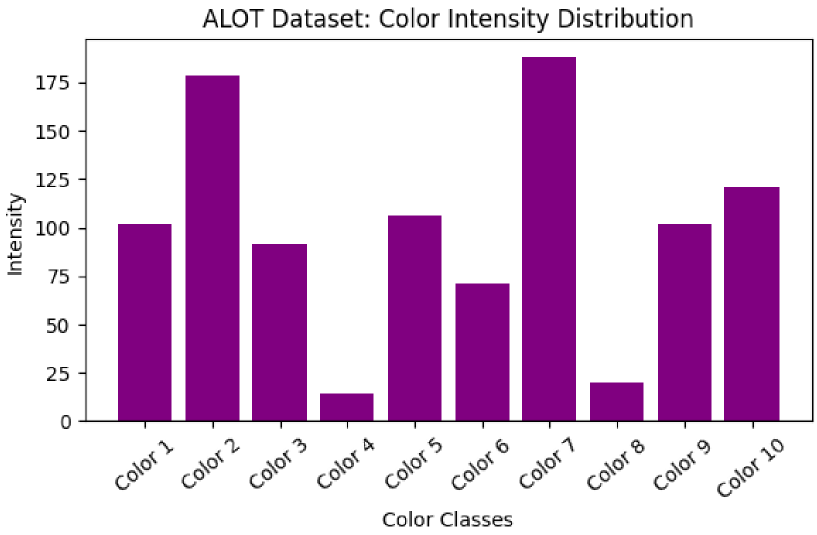

S6 Fig. Feature Distributions for ALOT Dataset

The feature distribution of Zubud, Tropical Fruits, Corel-10k, Cifar-10, and ALOT datasets is illustrated through the Scatter plot shown in S2 Fig, histogram, S3 Fig and S4 Fig, bar chart in S5 Fig and radar chart in S6 Fig. These are the facets of texture, shape and aspect ratio of the furniture.

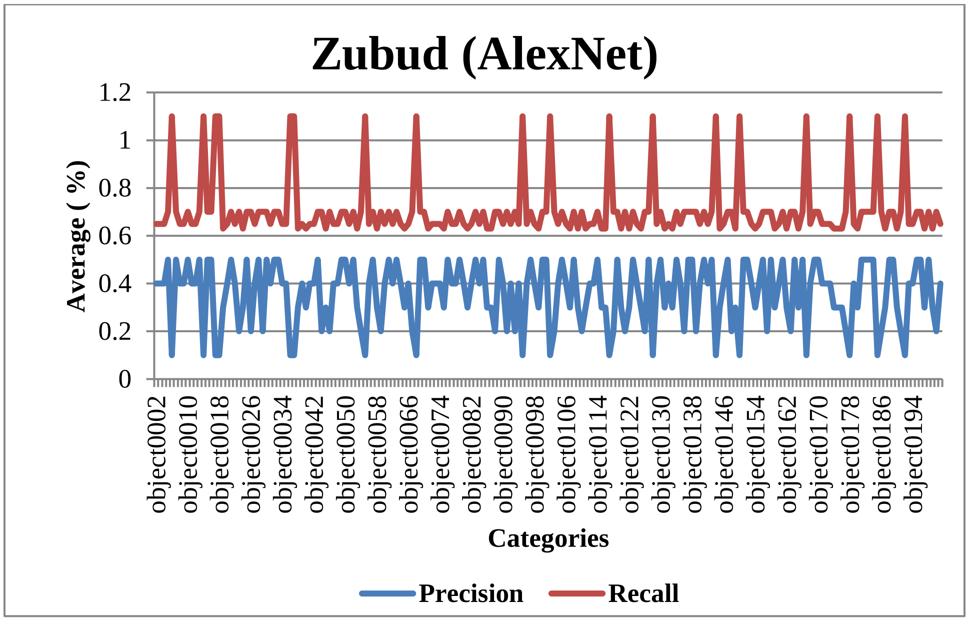

S7 Fig. Precision-Recall Curves for Zubud

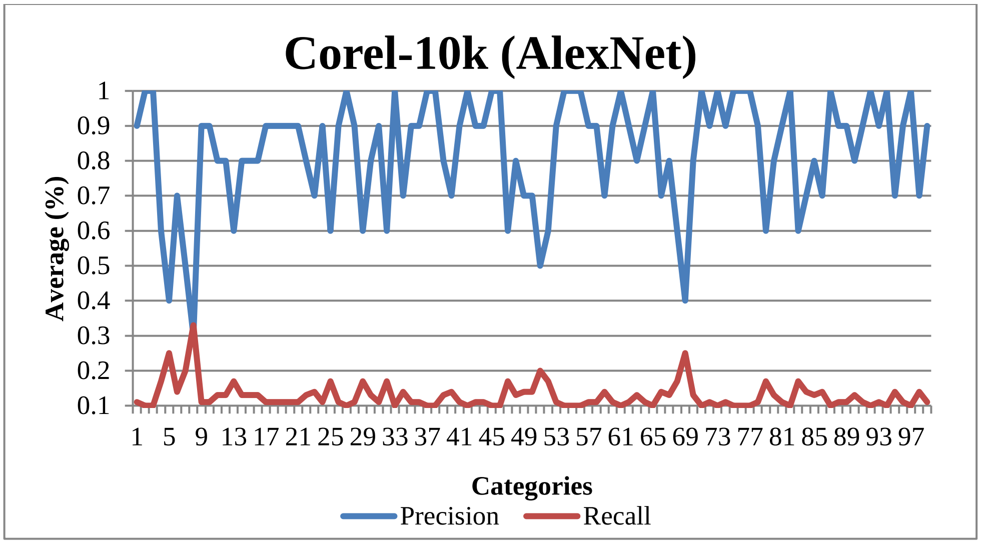

S8 Fig. Precision-Recall Curves for Corel-10k

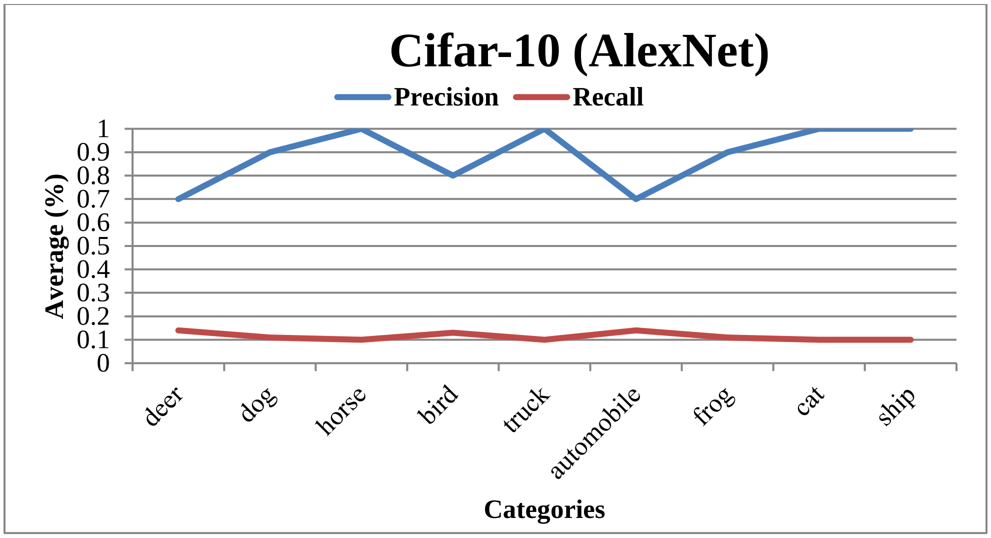

S9 Fig. Precision-Recall Curves for Cifar-10

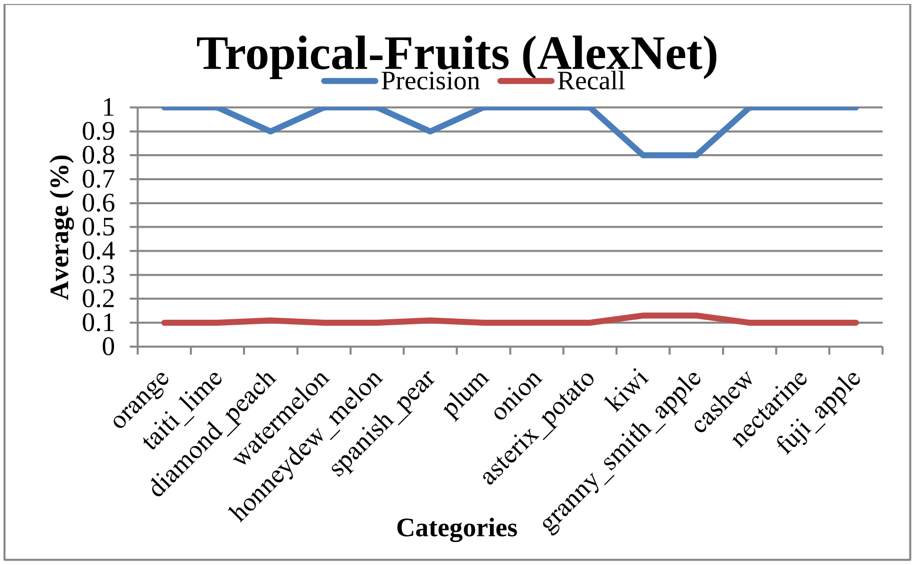

S10 Fig. Precision-Recall Curves for Tropical-Fruits

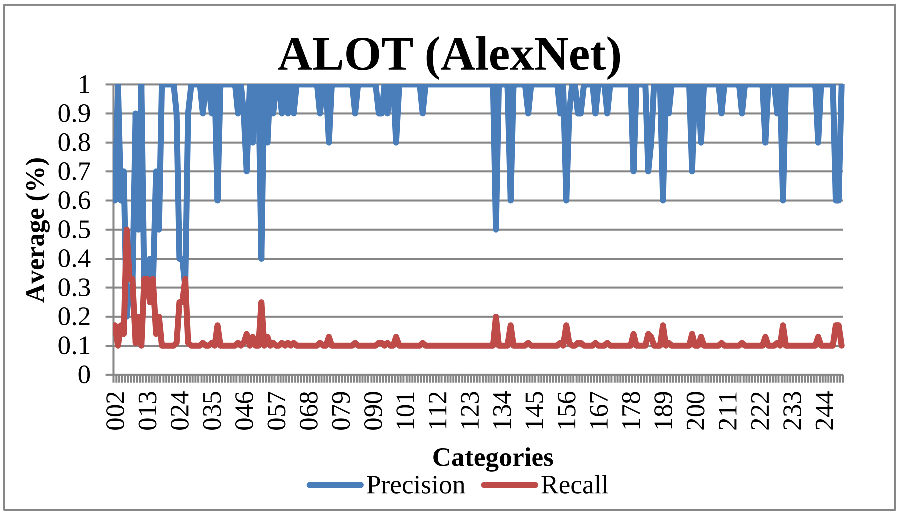

S11 Fig. Precision-Recall Curves for ALOT

S7 Fig, S8 Fig, S9 Fig, S10 Fig and S11 Fig representing precision-recall curves for proposed retrieval framework show that our framework can deliver high retrieval accuracy in different contexts.

2. Supplementary Tables

S1 Table. Dataset Characteristics

Dataset	Categories	Total Images	Key Features Covered
ALOT	250	25,000	Texture, color
Cifar-10	10	60,000	Shape, color
Corel-10k	100	10,000	Texture, spatial
Tropical Fruits	15	5,000	Shape, texture, color
ZuBuD	200	1,005	Complex objects, spatial features

Descriptive details of the datasets used to test the performance of CBIR approaches are presented in Table S1 to underscore the heterogeneity of the tested datasets.

S2 Table. Performance Metrics

Dataset	Precision (%)	Recall (%)	MAP (%)	Retrieval Time (ms)
ALOT	98.0	91.3	92.1	18.4
Cifar-10	97.0	88.2	88.8	21.7
Corel-10k	98.0	86.4	85.2	25.1
Tropical Fruits	99.0	93.8	93.6	16.2
Zubud	98.0	95.8	96.0	14.9

The performance evaluation for the proposed framework using different datasets is given in Table S2 in which the performances in terms of precision, recall and the mean average precision have increased.

3. Additional Methods and Details

Interest Point Detection

Suppression-Based Identification: Interest points are identified employing suppression schemes which calculate corner scores based on productive totals of the pixel first derivatives.

Scale-Space Interpolation: Interest points are then improved by adding weights of colour features normalized at the second level with the shape and objects.

Feature Extraction and Fusion

Traditional Features: Some high-variance coefficients are found that corresponds to dominating features. Some features are the color histograms, texture (for example GLCM) and shape.

Deep Features via CNNs: Features involve deeper levels by extracting features using a pre-trained CNN model which includes ResNet or Google Net. A multilevel fusion process is performed to integrate features from different layers to achieve higher discriminability.

Bag-of-Visual-Words (BoVW) Representation: A k-means clustering algorithm is then applied in order to clustered the feature vectors into visual words. For effective image searching and ranking process, BoVW representation is constructed.

Evaluation Metrics: The performance of the retrieval system is assessed using:

Precision: Total of images that were relevant to the topic.

Recall: Percentage of such images in the dataset identified.

Mean Average Precision (MAP): The average of the four precision scores amongst the four major categories.

4. Datasets and Benchmarks Information:

Dataset Details:

ALOT: The new texture dataset contains a a small set of 250 categories.

Source: Amsterdam Library of Textures (ALOT) - Homepage

Cifar-10: Standard dataset with 10 object classes for image classification task.

Source: http://www.cs.toronto.edu/~kriz/cifar.html.

Corel-10k: A group of datasets that can be used for any kind of CBIR task.

Source: GitHub - LionTao/CBIRDataset: Dataset module for SimpleCBIR

Tropical Fruits: Tropical Fruit Image Database for shape, color & texture.

Source: Custom-built.

ZuBuD: Exemplary case towards using complex objects and features of spatial forms in the development of Zurich constructing databases.

Source: https://icu.ee.ethz.ch/

5. Code and Reproducibility

Code and Data Availability: However the code and data for this study cannot be shared at the moment for [privacy reasons]. Nevertheless, the procedures and methods applied in this research have been declared which may help any other author to achieve comparable results in the like scenario. Both the code used in this work and the datasets that we used are available to the readers of this paper if they can get in touch with the corresponding author or the research team.

Methodology Overview:

To reproduce the experiments, follow these steps:

Dataset Preparation: First process the data to split the dataset into a training set, a validation set and a test set. Assure formatting of dataset to the standards stated in the paper: for example, dimension of images or labeling of particular images.

Feature Extraction: Understand fundamental ways of defining textures, colours & forms more especially conventionally. Use one of the simple approaches (for example, when implementing texture analysis based on GLCM or utilizing color histograms, or when identifying a complex model, such as deep learning, for feature extraction based on CNNs).

Model Training: Thus, you are also able to train your retrieval model by using the combined multilevel fusion of the traditional and the deep feature. In the case of CNNs, a pretrained CNN (ResNet or GoogleNet) is used, and the DL model is employed in feature extraction level. **Performance Evaluation:** Examples include precision, recall, mean average precision and the time taken by the system to get the documents. These metrics are described in the paper and can with a few line of code in MATLAB or Python be computed.

Steps for Replication:

Environment Setup: Ensure you have MATLAB (R2022b or later) or Python installed with the necessary libraries: **MATLAB:** These are MATLAB's toolkit: Image Processing Toolbox, Statistics and Machine Learning Toolbox.

Python: OpenCV, NumPy, PyTorch, Scikit learn.

Feature Extraction and Fusion: Use the feature extraction methods and fusion techniques described under Methods section to the scenes. This may entail application of both simple mathematical or deep learning for feature extraction processes.

Performance Metrics: Include functions to measure precision, recall as well as Mean Average Precision. Apply these functions using descriptions elicited from the paper.

Reproducibility of Results: By following the steps above, you should be able to generate comparably running results in the aspects of precision-recall curves, the calculation of the retrieval accuracy and other parameters. It's important not to mix the datasets or with similar features as ALOT, Cifar-10 datasets or any other equivalent data set.

Contact Information for Access

If you require the data for replication purposes, please contact:

Corresponding Author: Aiza Shabir, aiza.6322@wum.edu.pk

Institution/Organization: Bhauddin Zakariya University, Multan

6. References

1. Krizhevsky, A., & Hinton, G. (2009). Learning Multiple Layers of Features from Tiny Images. chromeextension://efaidnbmnnnibpcajpcglclefindmkaj/https://www.cs.utoronto.ca/~kriz/learning-features-2009-TR.pdf
2. Lowe, D. G. (2004). Distinctive Image Features from Scale-Invariant Keypoints. https://link.springer.com/article/10.1023/B:VISI.0000029664.99615.94
3. Babenko, B., & Lempitsky, V. (2015). Aggregating Local Deep Features for Image Retrieval. https://openaccess.thecvf.com/content_iccv_2015/html/Babenko_Aggregating_Local_Deep_ICCV_2015_paper.html
4. He, K., Zhang, X., Ren, S., & Sun, J. (2016). Deep Residual Learning for Image Recognition. https://openaccess.thecvf.com/content_cvpr_2016/html/He_Deep_Residual_Learning_CVPR_2016_paper.html

5. Tan, M., & Le, Q. V. (2019). EfficientNet: Rethinking Model Scaling for Convolutional Neural Networks. https://proceedings.mlr.press/v97/tan19a.html?ref=jina-ai-gmbh.ghost.io
6. Sivic, J., & Zisserman, A. (2003). Video Google: A Text Retrieval Approach to Object Matching in Videos. https://ieeexplore.ieee.org/abstract/document/1238663
7. Gonzalez, R. C., & Woods, R. E. (2008). Digital Image Processing (3rd ed.). https://books.google.com.pk/books?hl=en&lr=&id=a62xQ2r_f8wC&oi=fnd&pg=PA19&dq=7.%09Gonzalez,+R.+C.,+%26+Woods,+R.+E.+(2008).+Digital+Image+Processing+(3rd+ed.).+A+comprehensive+textbook+on+the+fundamentals+of+image+processing,+including+feature+extraction+and+image+analysis+techniques.&ots=3B2vL6jJ3J&sig=7ltUXyx0sVKAfMv_xJsQFkjU6j8&redir_esc=y#v=onepage&q&f=false.
8. Manning, C. D., & Schütze, H. (2008). Foundations of Statistical Natural Language Processing. https://books.google.com.pk/books?hl=en&lr=&id=YiFDxbEX3SUC&oi=fnd&pg=PR16&dq=8.%09Manning,+C.+D.,+%26+Sch%C3%BCtze,+H.+(2008).+Foundations+of+Statistical+Natural+Language+Processing.&ots=v0tinyfHSP&sig=pA_z2W-HCTIxOHHTQBFOH-A8nrA&redir_esc=y#v=onepage&q&f=false
9. Chum, O., & Matas, J. (2005). Matching with Prototypes. https://ieeexplore.ieee.org/abstract/document/5206529
10. Zhou, D., & Tian, Y. (2018). Multi-level Feature Fusion for Image Retrieval. https://link.springer.com/article/10.1007/s11554-019-00908-4
11. Zhou, B., & Wang, Y. (2017). Revisiting the Classification Framework for Image Retrieval with Deep Learning. https://www.sciencedirect.com/science/article/abs/pii/S0924271617303428
